# Supplementary material for: Layered BiOI single crystals capable of detecting low dose rates of X-rays
Source: Nat Commun. 2023 Apr 28;14:2452. doi: 10.1038/s41467-023-38008-4 (PMC10147687; doi:10.1038/s41467-023-38008-4)
Supplement: Supplementary file 1 — Supplementary Information [file 41467_2023_38008_MOESM1_ESM.pdf]

Supplementary Information for:

Layered BiOI single crystals capable of detecting low dose rates of X-rays

Robert A. Jagt[1†], Ivona Bravić[1,2†], Lissa Eyre[3,2], Krzysztof Gałkowski[2], Joanna Borowiec[4,5], Kavya Reddy Dudipala,[6] Michał Baranowski[7,8], Mateusz Dyksik[7,8], Tim W.J. van de Goor[2], Theo Kreouzis[4], Ming Xiao[1], Adrian Bevan[4], Paulina Płochocka[7,8], Samuel D. Stranks[2,9], Felix Deschler[3,2], Bartomeu Monserrat[1,2]*, Judith L. MacManus-Driscoll[1]*, Robert L. Z. Hoye[6,10]*

[1] Department of Materials Science and Metallurgy, University of Cambridge, 27 Charles Babbage Road, Cambridge CB3 0FS, UK

[2] Department of Physics, Cavendish Laboratory, University of Cambridge, 19 JJ Thomson Avenue, Cambridge CB3 0HE, UK

[3] Walter Schottky Institut, Technische Universität München, Am Coulombwall 4, Garching D-85748, Germany

[4] School of Physical and Chemical Sciences, Queen Mary University London, London E1 4NS, UK

[5] College of Physics, Sichuan University, Chengdu 610064, China

[6] Inorganic Chemistry Laboratory, Department of Chemistry, University of Oxford, South Parks Road, Oxford OX1 3QR, UK

[7] Laboratoire National des Champs Magnétiques Intenses, UPR 3228, CNRS-UGA-UPS-INSA, Grenoble and Toulouse, France

[8] Department of Experimental Physics, Wroclaw University of Science and Technology, Wroclaw, Poland

[9] Department of Chemical Engineering and Biotechnology, University of Cambridge, Philippa Fawcett Drive, Cambridge CB3 0AS, UK

[10] Department of Materials, Imperial College London, Exhibition Road, London SW7 2AZ, UK

[†] These authors contributed equally.

*Corresponding Authors, Email: bm418@cam.ac.uk, jld35@cam.ac.uk, robert.hoye@chem.ox.ac.uk

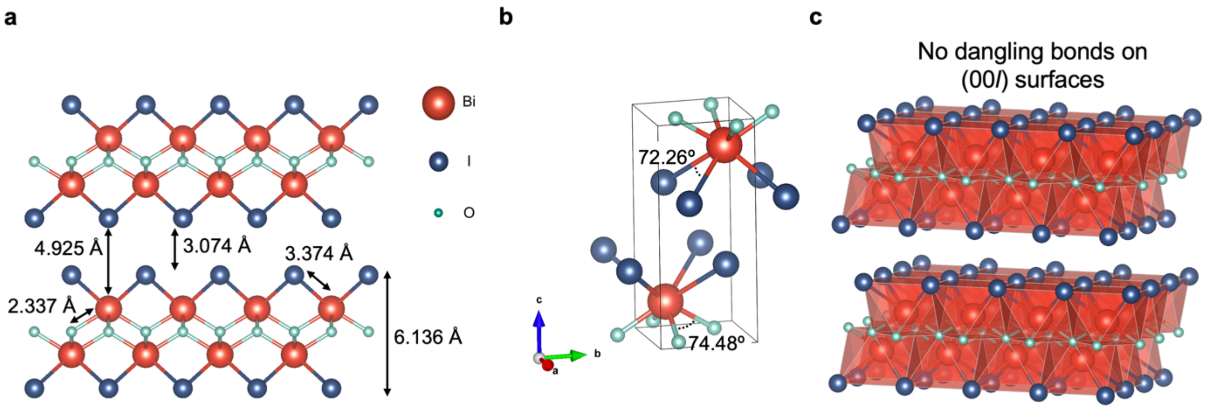

Supplementary Fig. 1 | Illustration of the crystal structure of BiOI determined experimentally. a, Two-dimensional view of the BiOI lattice, showing the bond lengths and inter/intra-layer distances obtained from refining the XRD data of single crystals ground to a powder sample. **b,** Unit cell of BiOI, with the bond angles illustrated. **c,** Three-dimensional illustration of two layers of the BiOI lattice, emphasising that the (00*l*) surface (*i.e.*, large flat surface of a BiOI crystal) would not have dangling bonds.

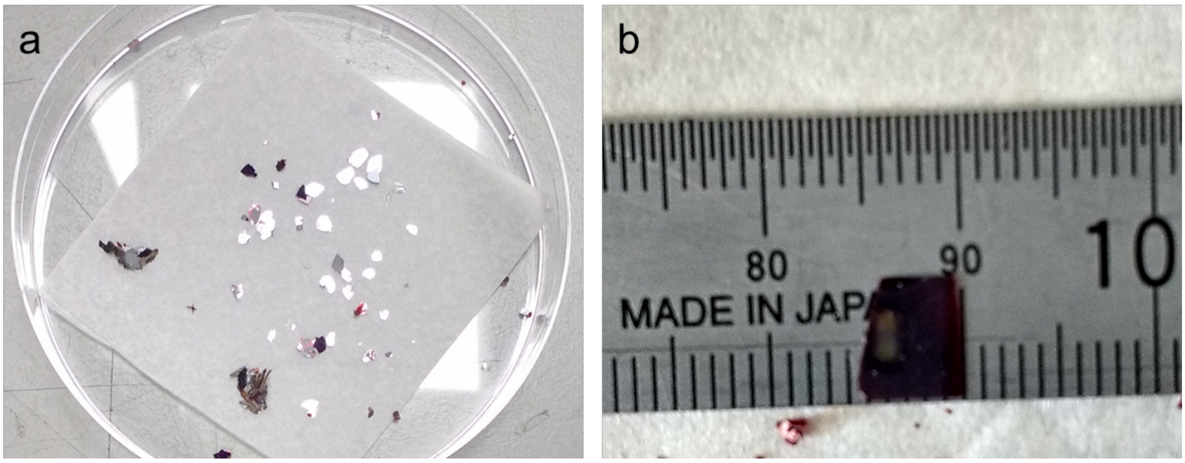

Supplementary Fig. 2 | Photographs of BiOI single crystals. a, Photograph of the result of a typical growth run of BiOI grown in sealed ampoules using chemical vapour transport (CVT). **b,** Example of BiOI crystal with dimensions. Typical crystals are several mm in size along the in-plane direction, and several hundreds of µm thick. The flat surface corresponds to the crystal (00*l*) face.

We crushed the single crystals to powder and performed powder X-ray diffraction. The refined parameters and measurement conditions are given in Supplementary Tables 1 and 2 below. The bond lengths, bond angles and intra-/inter-layer lengths are illustrated in Supplementary Fig. 1. Each I–Bi–O–Bi–I layer is thick, with a thickness of 6.136 Å, and the interlayer gap approximately half of this, at 3.074 Å. Whilst Bi–I bonds are covalent, we do not expect there to be strong interactions between Bi and the nearest I from the neighbouring layer. This is because: i) the distance between these atoms is large (4.925 Å), and ii) each layer is terminated with a layer of I spaced a unit cell apart each, and the large electron clouds around these I atoms would further Coulombically screen any interactions between Bi and the I from the neighbouring layer. Thus, BiOI should have *van der Waals* interactions between layers, and this has been found from several other studies[2–4]. Thus, in the single crystals, the (00*l*) surface would not have any dangling bonds (unless point or structural defects are introduced), which is beneficial for minimising surface recombination.

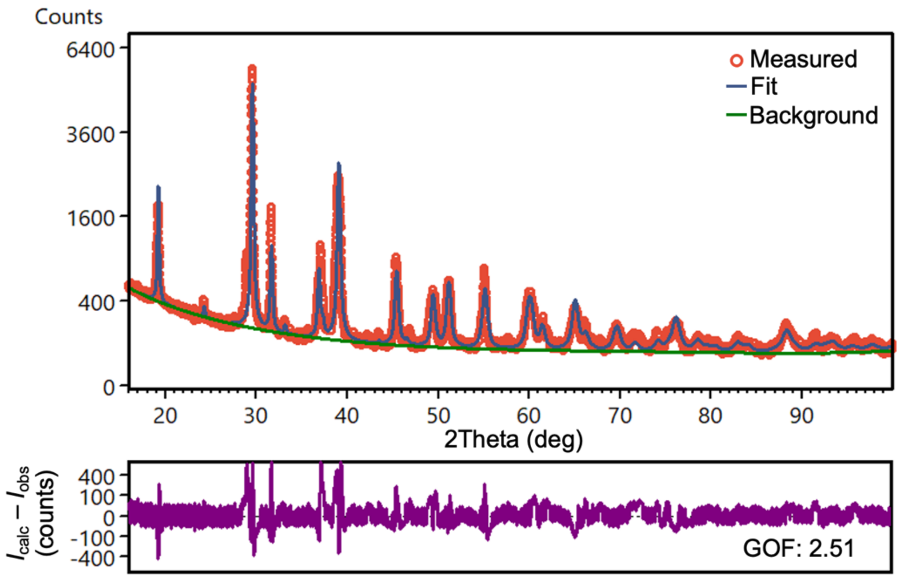

Supplementary Fig. 3 | Rietveld refinement of powder X-ray diffraction pattern from single crystals crushed to powder. The goodness of fit (GOF, χ) is shown, along with the residuals (purple), measured data points (red), fit (blue) and background (green).

Supplementary Table 1 | Data and structure refinement for BiOI single crystals measured in air under standard ambient laboratory lighting

Empirical formula	Bi O I
Molar mass	351.88 g mol^{-1}
Temperature	300 K
Wavelength	1.5406 Å
Crystal system, space group	Tetragonal, *P*4/*nmm*
Unit cell dimensions	*a* = 3.990 Å<br>*b* = 3.990 Å<br>*c* = 9.211 Å
Volume	146.61 Å^3
Z, Calculated density	2, 7.971 g cm^{-3}
Absorption coefficient	2500 cm^{-1}
F_{000}	288
θ range for data collection	16° to 100°
Step size, dwell time per step	0.01°, 1.5 s
Number of peaks	61
Refinement method	Rietveld
Goodness-of-fit, χ	2.51
$R_{expected}$	6.78%
R_{wp}	17.0%

Supplementary Table 2 | Atomic coordinates for BiOI, obtained from Rietveld refinement of powder sample (parameters given in Suppl. Table 1, fit in Suppl. Fig. 3).

Atom	*x*	*y*	*z*
Bi	1/4	1/4	0.1314
I	1/4	1/4	0.6661
O	1/4	3/4	0

Supplementary Table 3 | Bond lengths and angles for BiOI

Parameter	Value
Bi–O bond length	2.333 Å
O–Bi–O bond angle	74.40º
Bi–I (same layer)	3.382 Å
I–Bi–I bond angle	72.29º
Bi–I (different layers)	4.925 Å

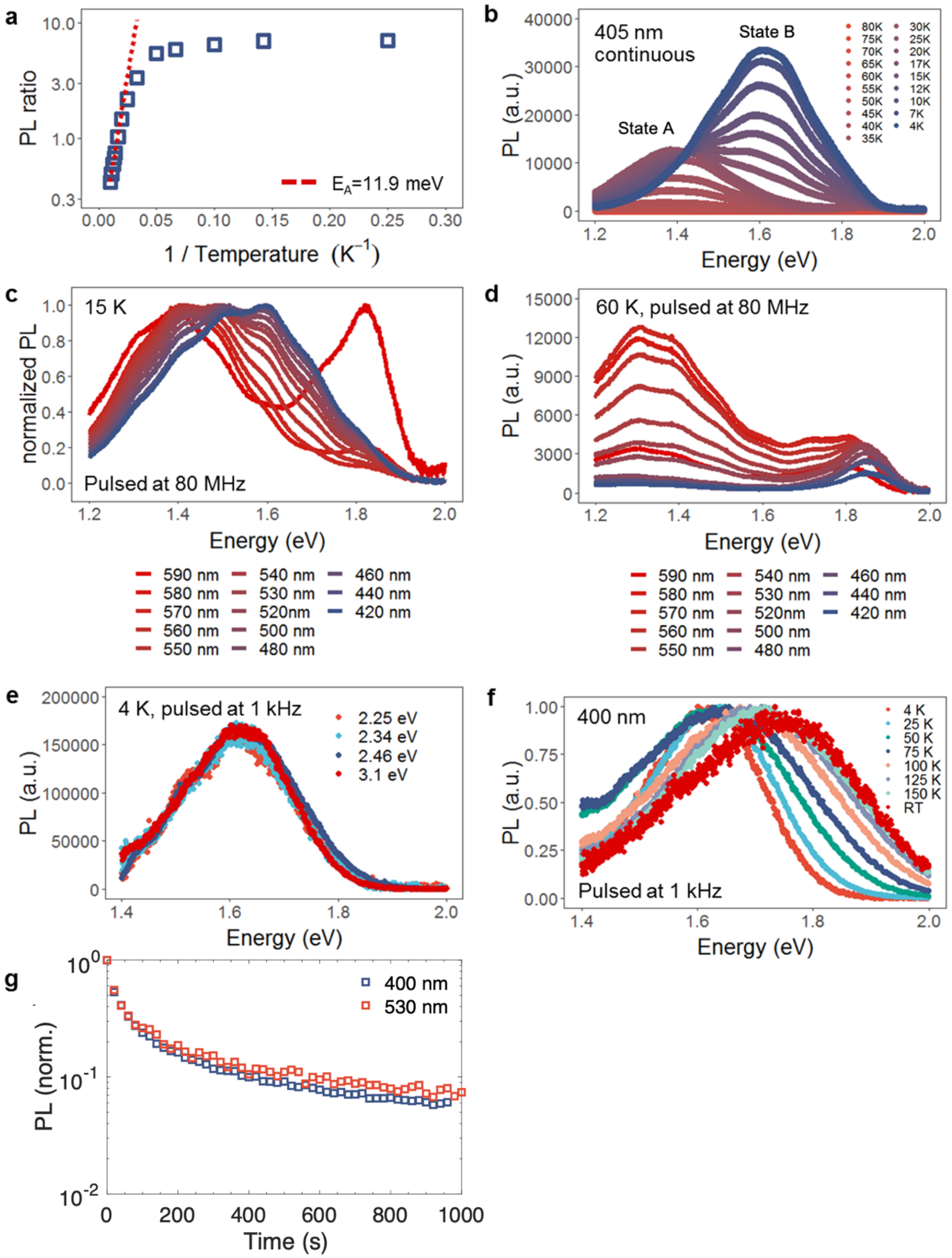

Supplementary Fig. 4 | Photoluminescence from BiOI single crystals. a, Ratio of the intensity of the PL peaks emitted around 1.3 eV (state A) and around 1.6 eV (state B) when illuminated with a continuous wave (cw) laser with wavelength of 532 nm as a function of inverse temperature. Here the PL ratio is defined as the maximum intensity at state B divided by the maximum intensity at state A. **b,** Temperature-dependent PL of a BiOI single crystal, illuminated with a cw 405 nm wavelength laser. Note the difference between the PL ratio of state A and state B in this figure and Fig. 1b of the main text. Excitation-dependent normalised PL of a BiOI single crystal measured at **c**, 15 K and **d**, 60 K,

illuminated with a pulsed laser with a repetition rate of 80 MHz. Note that the time in between pulses (12.5 ns at 80 MHz) is much shorter than the PL lifetime at cryogenic temperatures. Therefore, previously photo-excited carriers have not yet decayed. **e,** Excitation-dependent PL of a BiOI single crystal measured at 4 K, illuminated with a pulsed laser with a repetition rate of a 1000 Hz. **f,** Normalised temperature dependent PL of a BiOI single crystal, illuminated with a pulsed 400 nm laser, with a repetition rate of a 1000 Hz. Note that the time in between pulses (1 ms at 1 kHz) is much longer than the PL lifetime at cryogenic temperatures. Therefore, previously photo-excited carriers have almost all decayed before the next pulse arrives. **g,** Normalised time-resolved PL of a BiOI single crystal at 4 K, measured with an intensified charge-coupled device (iCCD) detector, and illuminated with a pulsed 400 nm and 530 nm laser. The repetition rate of the laser was 1 kHz. Both series depict similar decay profiles. This indicates that there is no to little effect caused by surface recombination at the crystal surface.

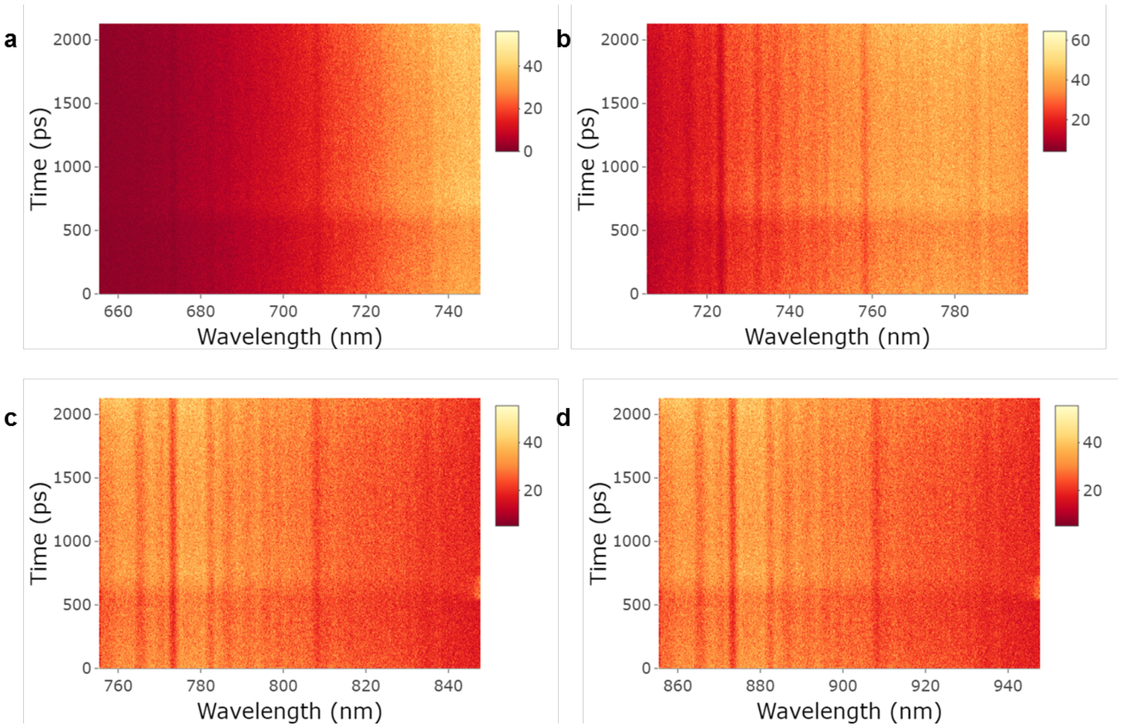

Supplementary Fig. 5 | PL decay of BiOI at 10 K measured using a streak camera. Measurements made with the spectrometer centred at **a,** 700 nm, **b,** 750 nm, **c,** 800 nm, and **d,** 900 nm wavelength. The excitation laser pulse (400 nm wavelength) was incident on the sample after a 500 ps delay time. The vertical lines present in the data are measurement artefacts.

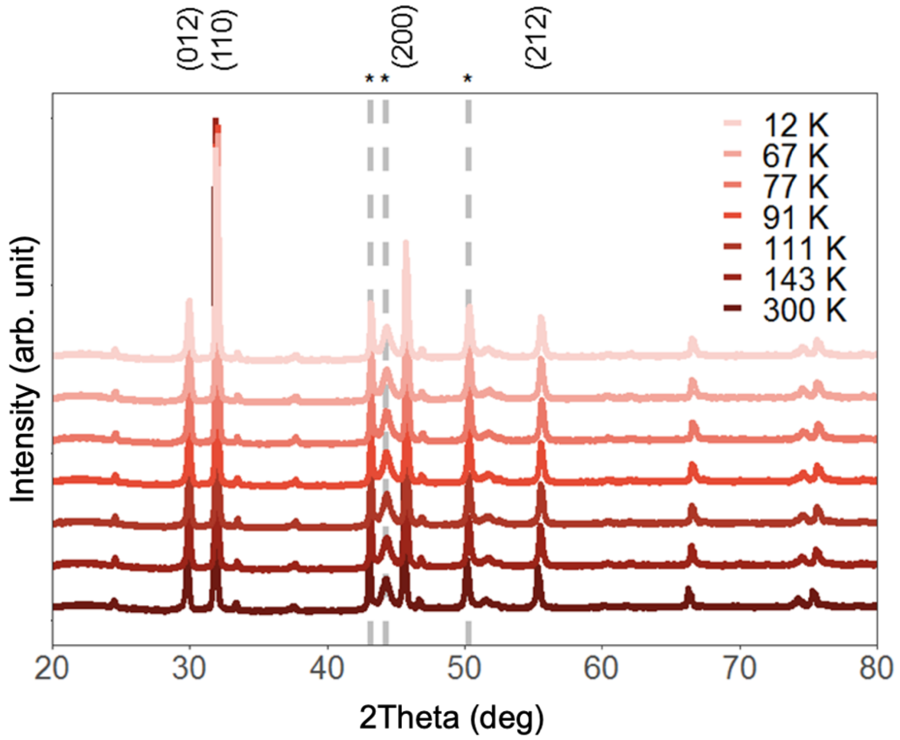

Supplementary Fig. 6 | Temperature-dependent X-ray diffraction pattern of BiOI thin films. * and grey dotted lines are peak from the substrates holder. No phase transitions are observed. Miller indices for BiOI are labelled on top. Rietveld refinement of these diffraction patterns shown in Supplementary Fig. 7 below, and the trends in fitted parameters in Supplementary Fig. 8.

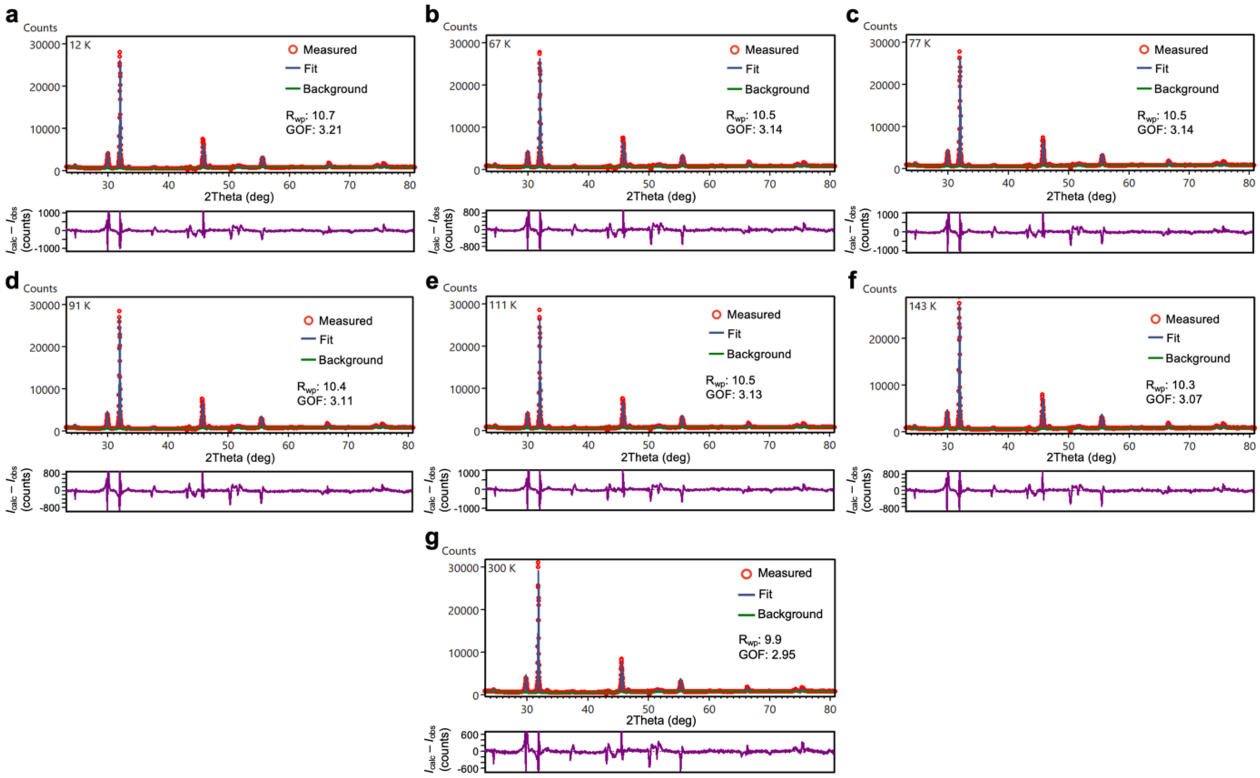

Supplementary Fig. 7 | Rietveld refinement of BiOI thin film X-ray diffraction patterns. Measured patterns (red), fits (blue), fitted backgrounds (green) and residuals for BiOI films at **a,** 12 K, **b,** 67 K, **c,** 77 K, **d,** 91 K, **e,** 111 K, **f,** 143 K and **g,** 300 K. The R_{wp} and goodness of fit (GOF, χ) are also shown.

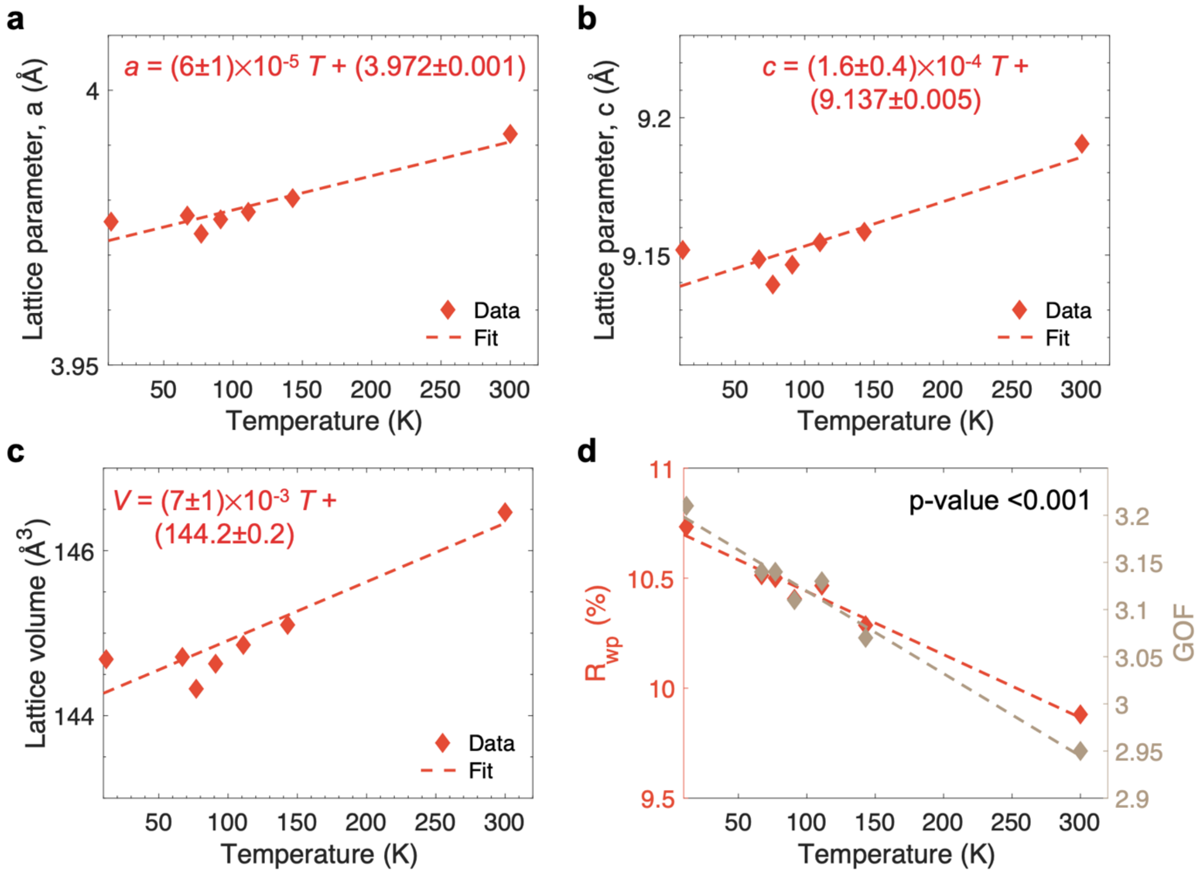

Supplementary Fig. 8 | Refined fitting parameters and agreement indices from temperature-dependent X-ray diffraction measurements on BiOI thin films. Fitted **a,** lattice parameter *a*, **b,** lattice parameter *c*, and **c,** unit cell volume of BiOI. **d,** R_{wp} and goodness of fit (GOF, χ) agreement indices. In parts **a**–**c**, the uncertainties shown are one standard deviation, and the *p*-values in all cases were <0.01, showing there to be strong evidence against the null hypothesis of no trend, *i.e.*, that these parameters do increase with temperature, as expected. In part **d**, the *p*-values were <0.001, showing there to be very strong evidence against the null hypothesis of temperature-invariance. This shows that the matlockite model for BiOI became a better fit for the long-range symmetry at higher temperatures. However, we note that this does not rule out the possibility of deviations from the matlockite structure in the short-range symmetry (<5 Å)[1].

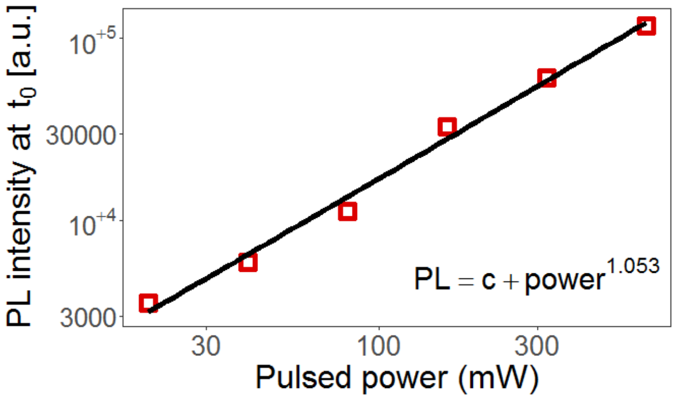

Supplementary Fig. 9 | Initial PL intensity after a laser pulse (400 nm) at a temperature of 4 K as a function of initial laser pulse power. The pulse frequency was 1000 Hz, such that all the PL decayed to zero before the next pulse arrived.

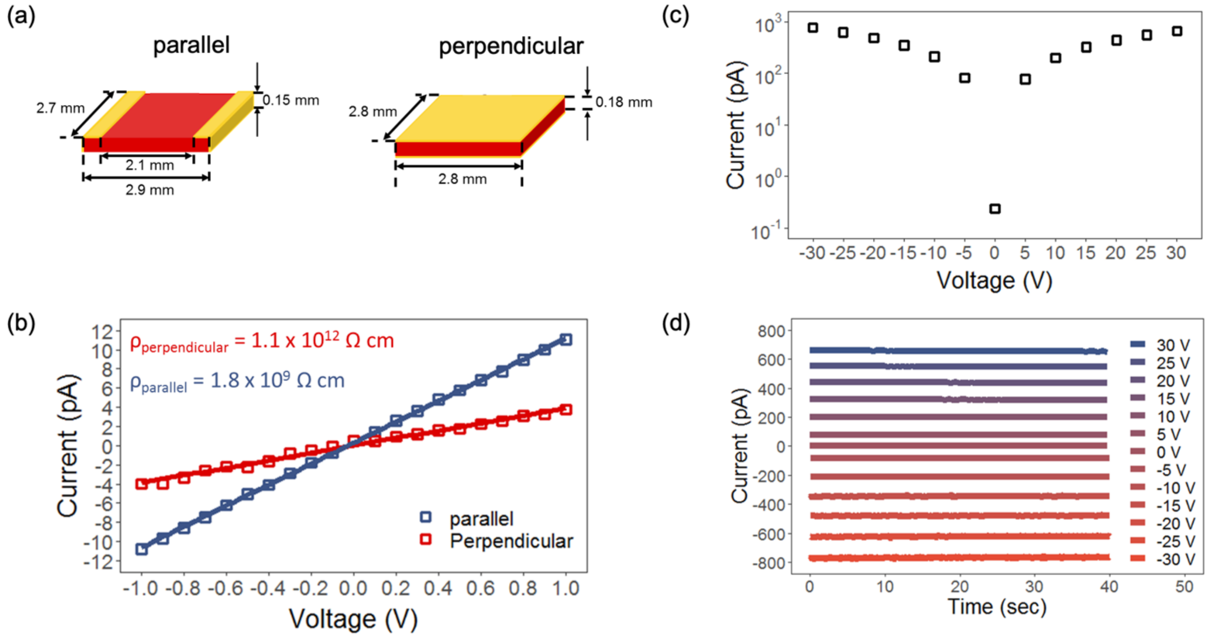

Supplementary Fig. 10 | Dark current measurements and device configurations of BiOI devices. a, Device dimensions for the parallel (left) and perpendicular (right) device configuration. **b,** Current-Voltage curves for devices in the parallel (blue) and perpendicular (red) device configurations. The resistivity values are depicted inset. **c,** Log-linear plot of the absolute value of the dark current of BiOI devices in the perpendicular configuration. **d,** Dark current of BiOI (perpendicular configuration) over time at different applied biases.

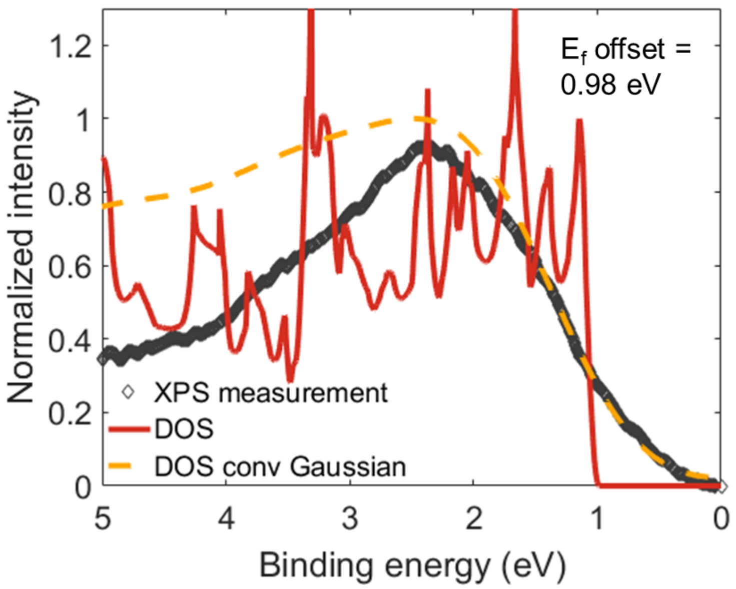

Supplementary Fig. 11 | Valence band spectrum of the X-ray photoemission spectroscopy spectrum of a BiOI single crystal. The fit with the density of states of BiOI was convoluted with a Gaussian to account for instrument broadening. The obtained Fermi-level offset to the valence band is 0.98 eV.

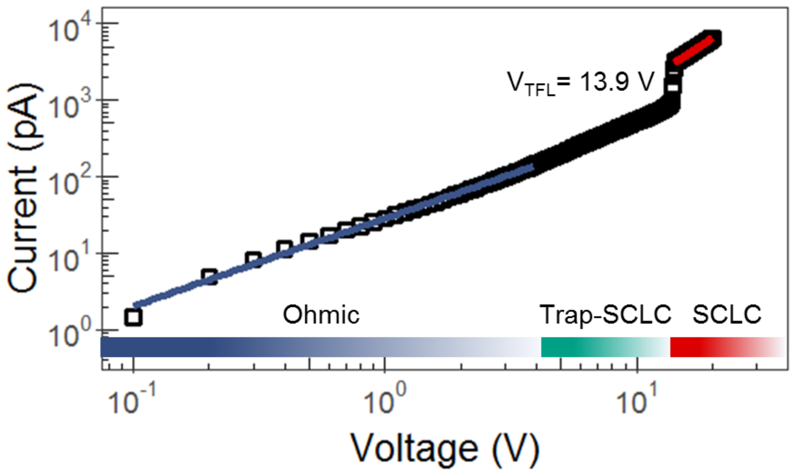

Supplementary Fig. 12 | Current-voltage curve for parallel BiOI devices. In these dark current measurements, there is a clear initial Ohmic regime, before entering into a space-charge regime after going through a non-linear regime (which can be due to the effects of shallow traps), and a steep trap filling regime. This is similar behaviour to previous SCLC measurements of high-mobility $MAPbI_3$ crystals[5]. In the BiOI crystals, the trap filled voltage is 13.9 V. The SCLC- mobility obtained was 54.3 V $cm^2 V^{-1}s^{-1}$, and the trap density was 2.3×10^9 cm^{-3}. The trap-filled voltage was selected to be the onset at which the current was proportional to V^n, where $n > 3$, and the SCLC regime where current has a quadratic dependence on voltage. Between the Ohmic regime and trap-filled voltage, there is a region labelled "trap-SCLC" because this is where the current has a non-linear dependence on voltage, which we believe to be due to the presence of traps that were not completely filled affecting the current. Please note that SCLC measurements of perpendicular BiOI devices are not shown because these did not enter into a clear space-charge regime.

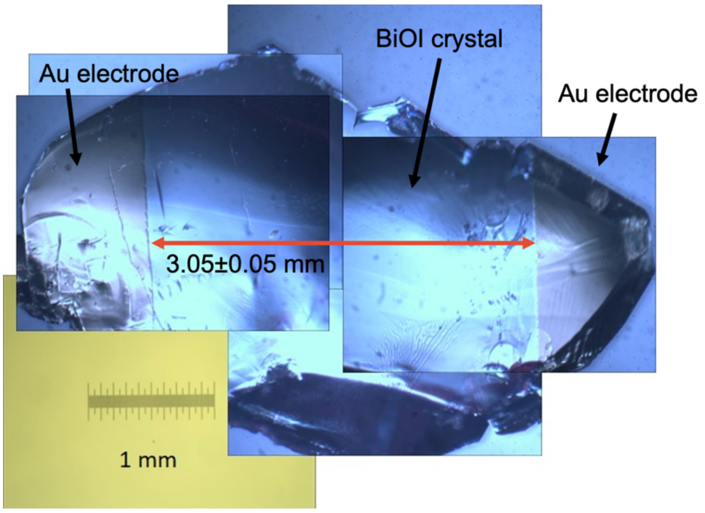

Supplementary Fig. 13 | Optical micrograph showing an example of a representative BiOI device made in the parallel structure. A composite of several micrographs was required because the device was too large in individual measurements. This device was different to the devices used for TOF and X-ray detector measurements, which had different dimensions. These dimensions were carefully measured using Vernier callipers. We carefully selected crystals that had the most rectangular shape, and the with a large central region bordered with parallel Au electrodes to enable a large region with well-defined electric field lines.

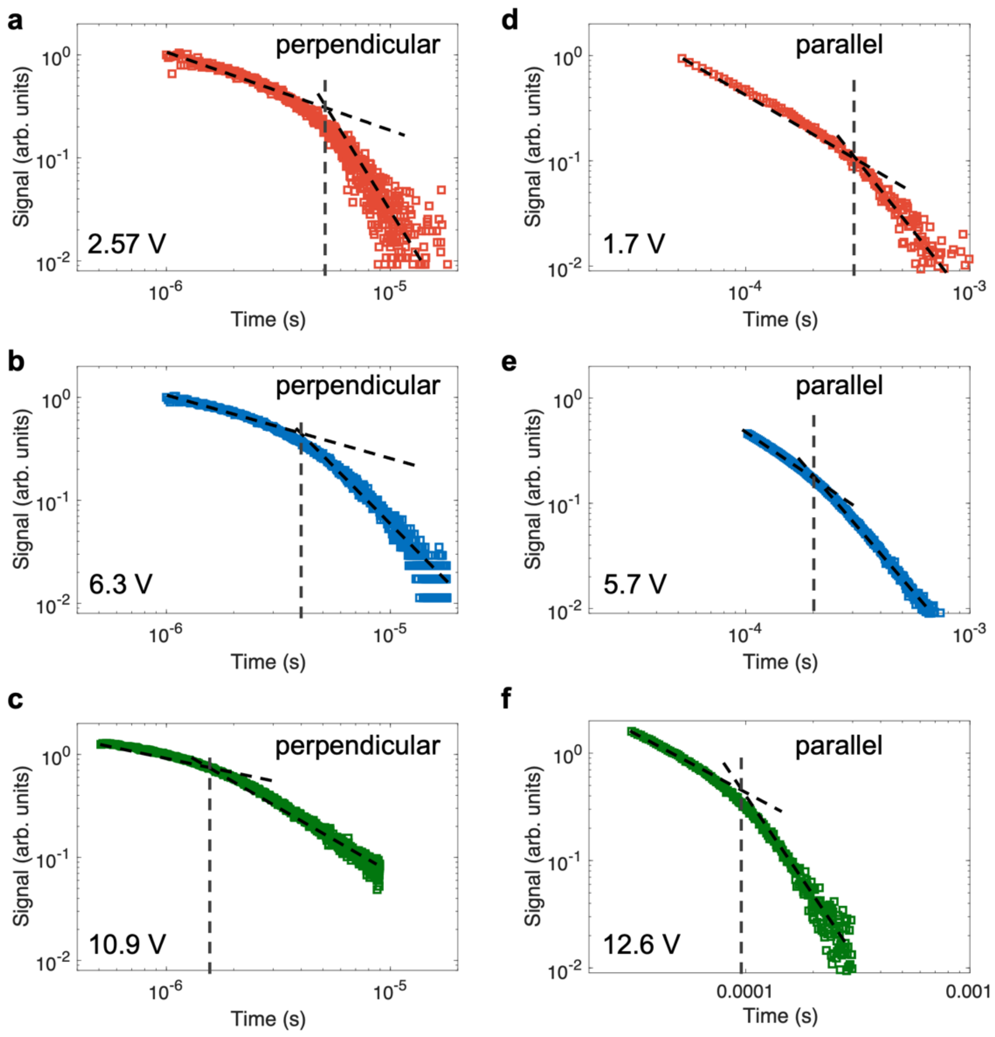

Supplementary Fig. 14 | Time-of-flight measurements for BiOI single crystal devices. Individual plots of the transient current curves in the (a–c) perpendicular, and (d–f) parallel configurations under different applied biases shown inset. Tangents fit, and their intersections to determine the arrival time (*t*) are indicated in dashed lines.

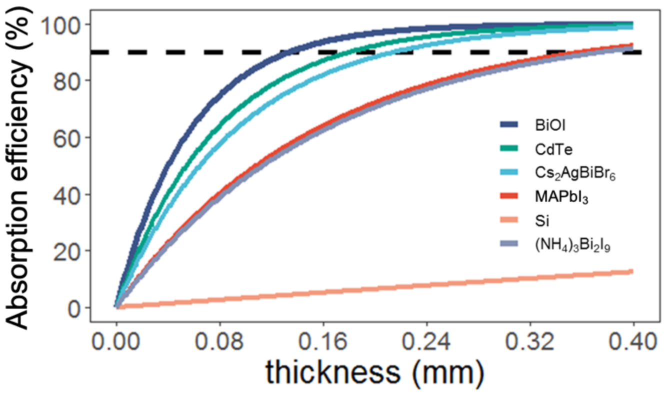

Supplementary Fig. 15 | Absorption efficiency of 30 keV X-rays as a function of material thickness, comparing BiOI, CdTe, $Cs_2AgBiBr_6$, $MAPbI_3$, $(NH_4)_3Bi_2I_9$, and Si. The dotted line indicates an absorption efficiency of 90%.

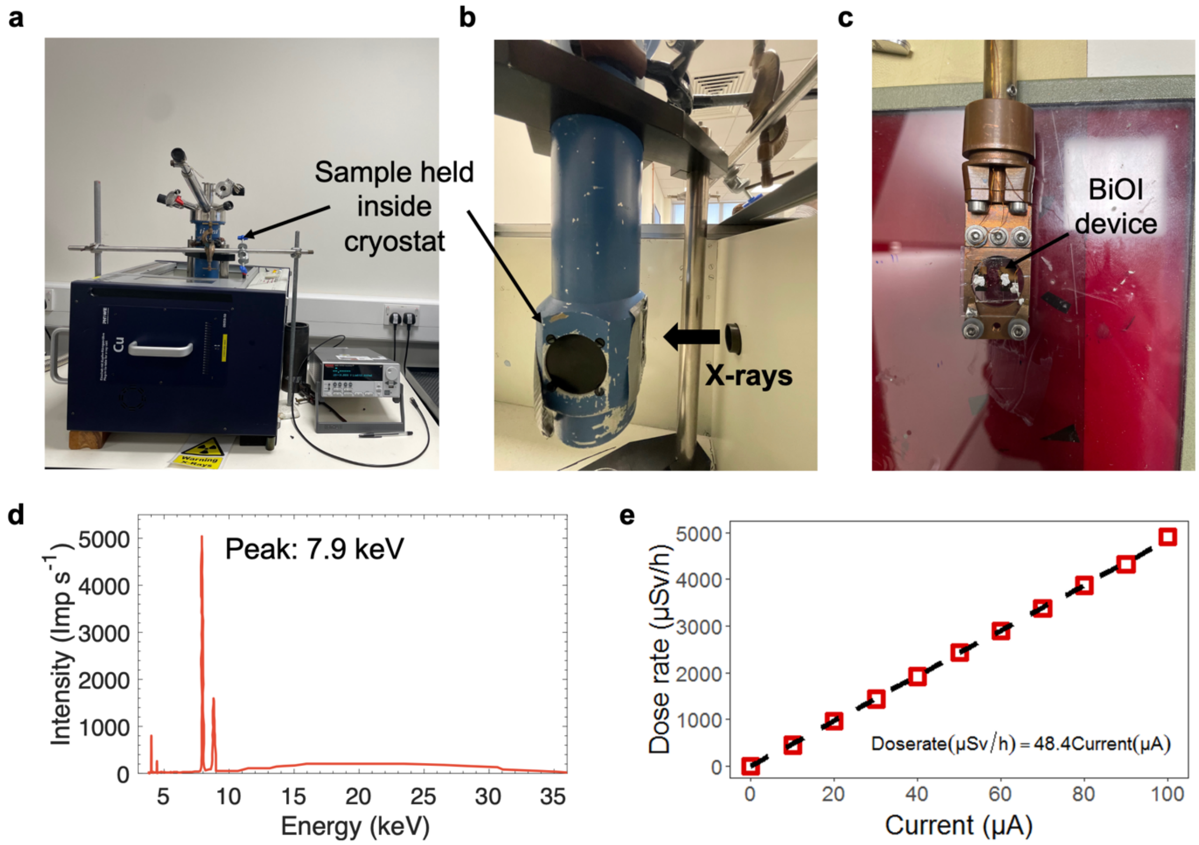

Supplementary Fig. 16 | Experimental details for X-ray detector measurements. a, Photograph of the whole setup, including the X-ray source and SMU for measuring the photocurrent. **b**, Photograph inside the measurement chamber, showing how the X-ray source is aligned with the location of the sample inside the cryostat. The window of the cryostat is covered with dark tape to prevent illumination from visible light. All samples were mounted in the same way to the cryostat, which was fixed in position. Thus, any X-ray ionisation of air or reflection effects, which we expect to be small, would be the same for all samples. **c**, Photograph of the X-ray detector device mounted onto a glass substrate, and connected via wires to the electrical connections on the cryostat holder. **d**, Spectrum of the X-ray source **e**, Calibration curve for the X-ray source, correlating the dose rate (measured with an ion chamber) with the current provided to the X-ray tube.

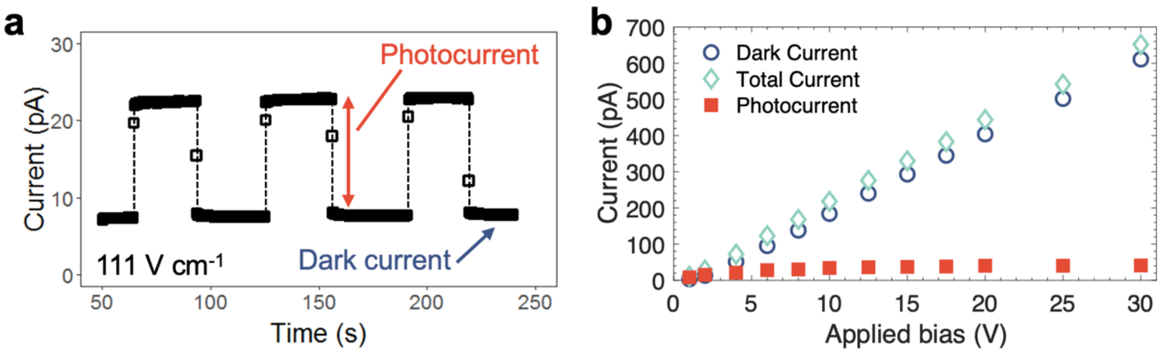

Supplementary Fig. 17 | Photocurrent and dark current from BiOI devices in the perpendicular configuration. a, Illustration of the photo- and dark current, and **b**, comparison of the photo-, dark and total (photocurrent + dark current) as a function of applied bias. Whilst the photocurrent initially exceeds the dark current at low applied biases, the photocurrent saturates (since photocarriers are

only generated at one electrode), whilst the dark current continues to rise (since these carriers are available in the entire bulk of BiOI) with increasing applied bias.

Supplementary note 1 | Effective atomic number (Z_{eff}) and absorption coefficient calculations

The effective atomic number of non-binary semiconductors is calculated using a non-linear relationship between the effective atomic mass and the atomic mass of the constituent atoms:

$$Z_{\mathrm{eff}} = \sqrt[n]{f_1.(Z_1)^n + f_2.(Z_2)^n + \cdots + f_k.(Z_k)^n} \quad \text{(S1)}$$

where f_m is the fraction of the total number of electrons associated with each element, Z_m is the atomic number of each element m, and n is a number (here taken to be 2.94). The calculations of the effective atomic numbers can be found in "Effective Z number.m" in the associated research data repository for this paper; please refer to the Data availability statement in the main text.

This power law method is only a first order approximation[6] and it is more appropriate to calculate the absorption profile due to the several processes that can lead to photon absorption. These processes are the photoelectric effect, coherent Rayleigh scattering, incoherent Compton scattering, electron-positron production, or photonuclear reactions. The absorption profile are calculated using open source software[7] provided by the National Institute of Standards and Technology, called: "NIST Standard Reference Database 8 (XGAM)".

Supplementary note 2 | Model describing the optical transmission through BiOI single crystals

The transmission through BiOI single crystals is modelled using the transfer-matrix method[8]. For schematic of the modelled situation see Supplementary Fig. 18 below.

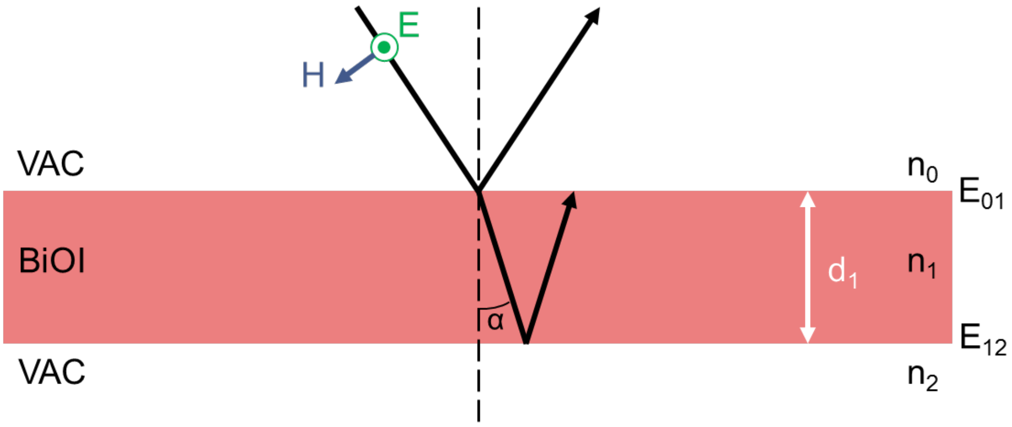

Supplementary Fig. 18 | Schematic indicating the modelled situation of light passing from vacuum, through the BiOI single crystal, to the other side of the crystal. Here n_i represents the index of refraction of the ith layer, d_1 the crystal thickness, E the electric polarisation, H the magnetic polarisation, E_{01} the tangential component of the electric field going from vacuum into the BiOI crystal, E_{12} the tangential component of the electric field going from the BiOI crystal into vacuum, and α the angle that the light makes with respect to the crystal normal.

The transfer matrix method connects the tangential field entering the crystal to the tangential field exciting the crystal. Mathematically this can be written as

$$\begin{pmatrix} E_{01} \\ \eta_0 H_{01} \end{pmatrix} = \begin{pmatrix} \cos\delta_1 & \frac{i\sin\delta_1}{\gamma_1} \\ i\gamma_1 \sin\delta_1 & \cos\delta_1 \end{pmatrix} \begin{pmatrix} E_{12} \\ \eta_0 H_{12} \end{pmatrix} = M \begin{pmatrix} E_{12} \\ \eta_0 H_{12} \end{pmatrix} \tag{S2}$$

where $\gamma_1 = n_1 \cos\alpha_1$, $\delta_1 = \frac{2\pi}{\lambda} n_1 d_1 \cos\alpha_1$, and λ is the wavelength.

The reflection and transmission amplitude r and t are then given by

$$r = \frac{\gamma_0 m_{11} + \gamma_0\gamma_2 m_{12} - m_{21} - \gamma_2 m_{22}}{\gamma_0 m_{11} + \gamma_0\gamma_2 m_{12} + m_{21} + \gamma_2 m_{22}} \tag{S3}$$

$$t = \frac{2\gamma_0}{\gamma_0 m_{11} + \gamma_0\gamma_2 m_{12} + m_{21} + \gamma_2 m_{22}} \tag{S4}$$

The index of refraction of the BiOI single crystal (layer 1) is complex valued; $n_1 = \underline{n_1} + \underline{k_1} i$. Here $\underline{k_1}$ is given by $\underline{k_1} = \frac{-\alpha\lambda}{4\pi} = \frac{-\lambda}{4\pi}\alpha_0 \exp\left(\frac{E-E_0}{E_\mathrm{u}}\right)$, where α is the absorption coefficient, E_0 and α_0 are constants, and E_u is the Urbach energy. For transmission parallel to the crystal surface normal, we have α = 0, giving $\gamma_1 = n_1$, $\delta_1 = \frac{2\pi}{\lambda} n_1 d_1$. The reflection and transmission amplitude r and t are then given by:

$$r = \frac{\left(\frac{1}{n_1} - n_1\right) i \sin\left(\frac{2\pi}{\lambda} n_1 d_1\right)}{\left(\frac{1}{n_1} + n_1\right) i \sin\left(\frac{2\pi}{\lambda} n_1 d_1\right) + 2\cos\left(\frac{2\pi}{\lambda} n_1 d_1\right)} \tag{S5}$$

$$t = \frac{2}{\left(\frac{1}{n_1} + n_1\right) i \sin\left(\frac{2\pi}{\lambda} n_1 d_1\right) + 2\cos\left(\frac{2\pi}{\lambda} n_1 d_1\right)} \tag{S6}$$

Finally, the transmission and reflection intensity T and R are given by $T = |t|^2$ and $R = |r|^2$.

An example of the transmission and the model fit is depicted in Supplementary Fig. 19.

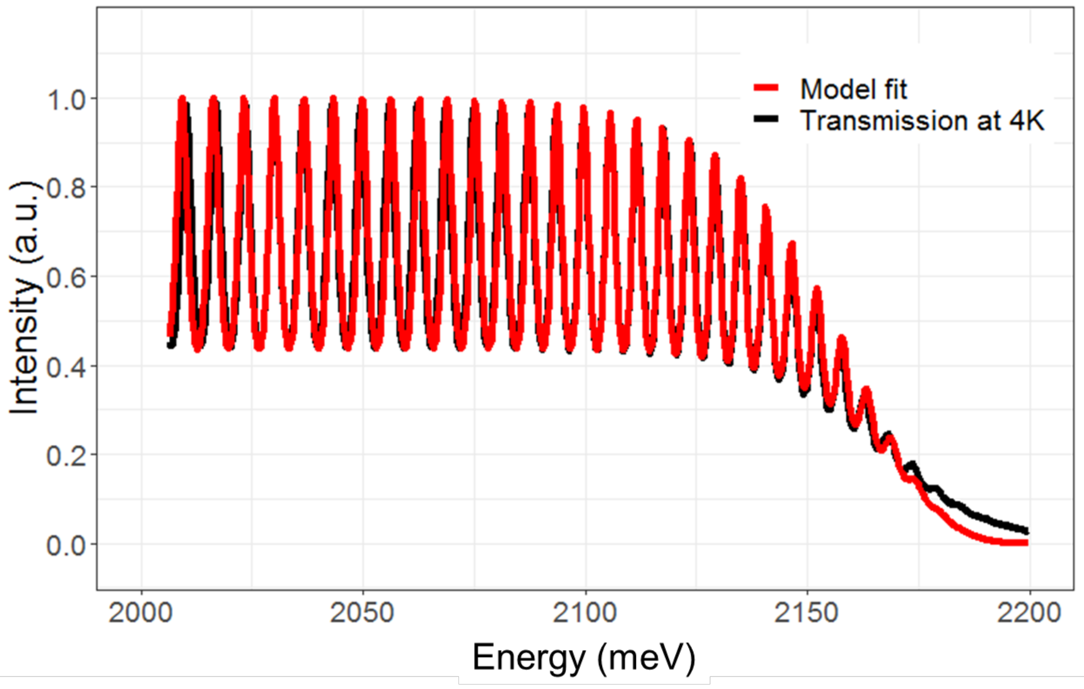

Supplementary Fig. 19 | UV-vis data of BiOI single crystal at 4 K. The optical transmission of BiOI single crystals measured using UV-vis (black), together with a fit based on the optical model (red) described in this section.

Supplementary note 3 | Calculations on thermodynamic defect concentrations

The thermodynamic defect concentration is based on a simple model and is calculated for various formation enthalpies. Contribution of the formation entropy to the defect concentration in equilibrium is omitted.

The number of defect sites N_s can be approximated as:

$$N_\mathrm{s} = \frac{N_A\,\rho_\mathrm{BiOI}}{A_\mathrm{BiOI}} \tag{S7}$$

where N_A is the Avogadro constant, ρ_BiOI is the mass density of BiOI, and A_BiOI is the atomic mass of BiOI.

Under thermodynamic equilibrium, the defect concentration N_defects can be approximated as

$$N_\mathrm{defects} = N_\mathrm{s} \exp\left(\frac{-\Delta H_\mathrm{f}}{k_\mathrm{b} T}\right) \tag{S8}$$

where ΔH_f is the formation enthalpy of the defect, k_b is the Boltzmann constant, and T is the temperature.

Upon cooling of the crystals, the equilibrium concentration of defects decreases. However, it depends on the diffusion kinetics of the vacancy, interstitial, and anti-site defects whether this equilibrium is reached. When cooled too fast defects get "frozen in" yielding a higher defect concentration than when cooled under thermodynamic equilibrium conditions (Supplementary Fig. 20). The distance the defects need to diffuse during the cooling down period to obtain thermodynamic equilibrium conditions is dependent on the defect concentration and is depicted in Supplementary Fig. 21. The diffusion constants of atoms in BiOI are unknown. Previously the it was found that the halide atoms in one-dimensional lead halide perovskite nanowires can diffuse 10 μm in 10 h at 100 °C.[9]

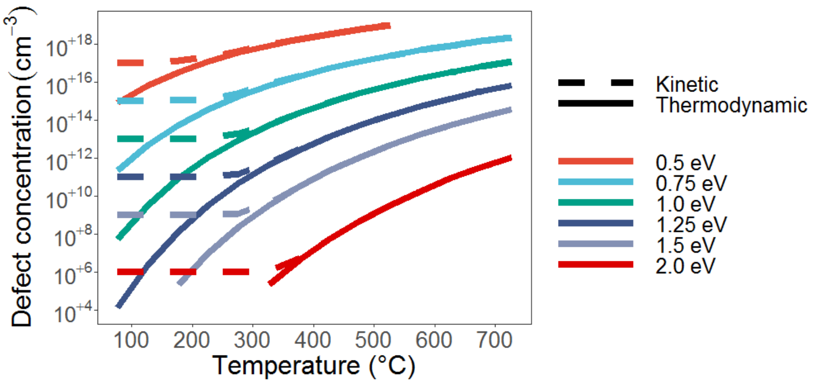

Supplementary Fig. 20 | Defect concentrations in BiOI as a function of temperature. Upon cooling the thermodynamic defect concentration (solid line) and the kinetically controlled defect concentration (dashed line) drop. Under kinetically controlled conditions defects can get frozen in, increasing the defect concentration.

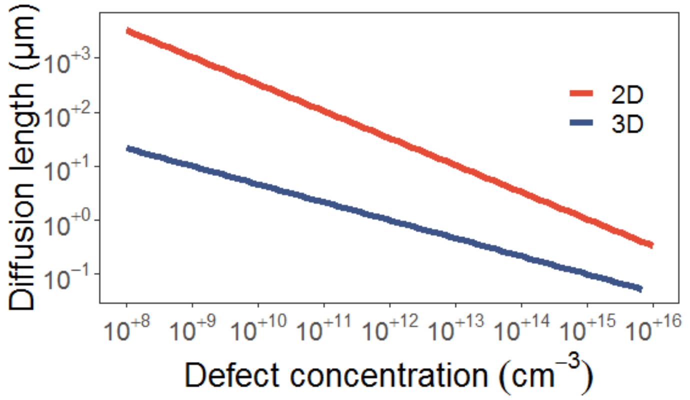

Supplementary Fig. 21 | Required diffusion length of atoms as a function of defect concentration. The required diffusion length is plotted both under the assumption of isotropic diffusion in two dimensions (red) and three dimensions (blue).

Supplementary note 4 | PL Quenching model

A simple phonon-mediated quenching model can be used to analyse the temperature-dependence of the PL intensity. Phonons can cause quenching of the PL by scattering it into a non-radiative state by coupling the carrier directly to the ground state. If the rate of non-radiative decay, k_{nr}, depends on the density of phonons N_{ph} as $k_{\mathrm{nr}} = k_{\mathrm{nr,0}} N_{\mathrm{ph}}$, and assuming a fixed radiative rate, k_{r}, then the PLQE becomes,

$$\mathrm{PLQE} = \frac{k_r}{k_r + k_n} = \frac{1}{1+\frac{k_{nr,0}}{k_r} N_{ph}(T)} \tag{S9}$$

Therefore, the temperature dependence of the emitted intensity, I, is,

$$\frac{I(T)}{I_0} = \frac{1}{1+\frac{k_{nr,0}}{k_r} N_{ph}(T)} \tag{S10}$$

where I_0 is the PL intensity at 0 K and can be estimated by extrapolation. The number of phonons populated at a certain temperature follow the Maxwell–Boltzmann statistics. Therefore, the above relationship in Equation S10 can be rewritten to:

$$\log\left(\frac{I_0}{I} - 1\right) = \frac{-E_A}{k_b T} + c \tag{S11}$$

where c is a constant, and E_A is the activation energy for non-radiative decay of the PL.

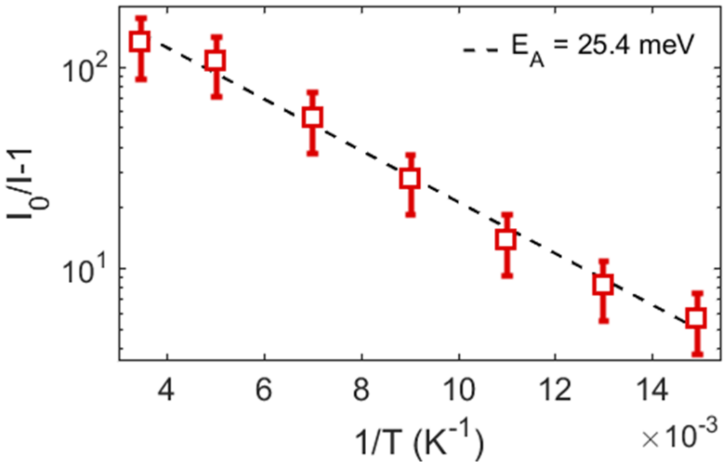

Supplementary Fig. 22 | Relationship between temperature and total integrated PL intensity. From the formula above an activation energy of 25.4 meV for non-radiative decay is obtained.

The PL spectrum depicted in Fig. 1b of the main text is composed of multiple emissive states, where the PL ratio of the emissive states is temperature dependent (Supplementary Fig. 4a). This phenomena has been observed in many materials, which arises due to thermally-driven back transfer between the two states[10,11]. In Supplementary Fig. 23, a schematic representation is depicted of the potential energy landscape. A carrier in the ground state can absorb a photon and be photoexcited (1). This photoexcited carrier can cause multiple structural deformations of the lattice, here labelled states A & B. These states are at different energy levels (difference of E_{ST}) separated by an energy barrier. The observed ratio of luminescence originating from state A, I_A, and state B, I_B, can then be written as[11]

$$\left(\frac{I_A}{I_B}\right)_{obs} = R \exp\left(\frac{-E_{ST}}{k_b T}\right) \tag{S12}$$

where R is a factor taking into account the optical transition probabilities and occupation of both states. Based on this equation the separation between the energy states was estimated to be 11.9 meV (Supplementary Fig. 4a).

Under continuous illumination, the equilibrium occupation of state A and B can be controlled through the excitation wavelength (Fig. 1b, main text, Supplementary Fig. 4b, 4c and 4d). States A and B correspond to the situation where the lattice distorts along one, or both dominant optical phonon modes. The excitation wavelength can control the direction of this distortion and therefore control the intensity ratio of both states. Furthermore, close to the transition temperature, where the non-radiative decay pathway (3) starts to dominate over the radiative transition (2), the non-radiative decay of state B can also be controlled through the excitation wavelength. Photoexcitation with higher-energy (*i.e.*, bluer) photons results in extra phonons being introduced to the lattice, thus promoting a greater fraction of charge-carriers to relax into State B, and further undergo process 3 to non-radiatively decay into the ground state.

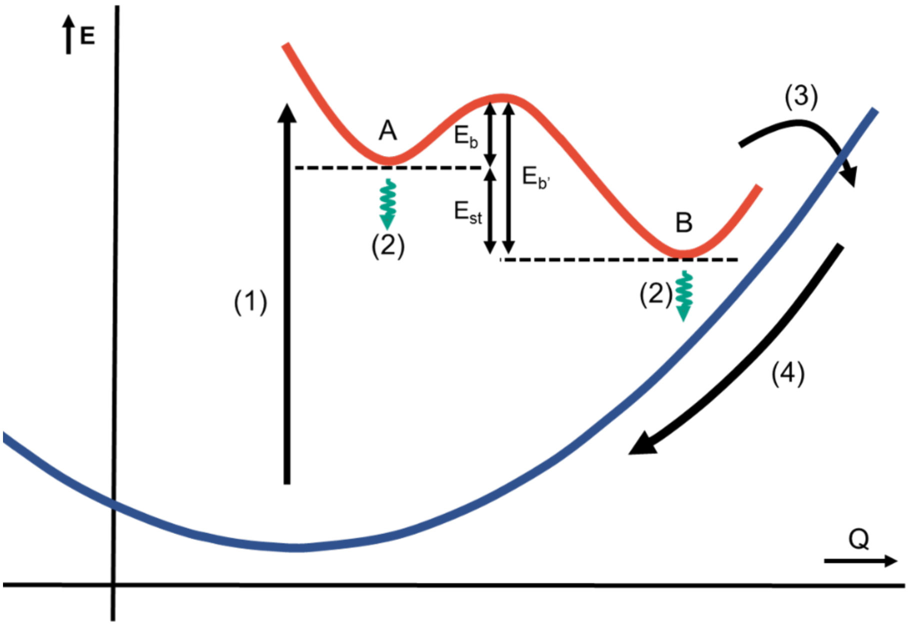

Supplementary Fig. 23 | Schematic representation of potential energy landscape of two emissive states. The states A & B are separated by an energy barrier and are at different energies, as depicted.

Supplementary note 5 | Note on transient absorption measurements and the Fourier transform of oscillating component

Coherent oscillations in the magnitude of the differential transmission, $\Delta T/T$, were observed at delay times up to 5 ps (Fig. 1d in the main text). In order to compare the frequency of these oscillations, a multiexponential decay curve was subtracted from each wavelength, leaving an oscillating residual centred on $\Delta T/T$=0. This residual was then Fourier transformed to give the power spectrum of oscillating frequencies. The frequency, ν, is expressed in wavenumbers, $1/\lambda$, according to the relation $1/\lambda=\nu/c$, where c is the speed of light in a vacuum. These spectra are depicted in Fig. 1e and Fig. 1f of the main text.

The possible origins of transient oscillations in the pump-probe signal, corresponding to Raman-active phonon frequencies, are 1) impulsive stimulated Raman scattering (ISRS), which does not require electronic transitions to occur, or 2) impulsive absorption, which requires the photoexcitation of charge-carriers to initiate certain coherent phonons. The absence of the 50 cm^{-1} Raman-active modes in the TA oscillations excludes ISRS as a mechanism for producing the oscillations and is consistent with the displacive excitation of coherent phonons.

The ratio of peak heights is more similar to the resonant Raman than the non-resonant Raman measurements, indicating that the excited state is coupled to the phonons (Fig. 1e in main text). Furthermore, when a sub-band gap pump was used for TA measurements with a fluence of 1 mJ cm^{-2} pulse^{-1} (chosen to be a high fluence below the threshold for two-photon absorption), no oscillations were observed in the probe transmission. Together with the previous observations, this points towards an impulsive absorption mechanism for generating the coherent phonons and that the lattice distorts along the two intralayer breathing modes after photoexcitation.

Supplementary note 6 | Temperature dependent Urbach absorption edge

The exponential tail in the below band gap absorption tail can be fitted by the Urbach model

$$\alpha(E,T) = \alpha_0 \exp\left[\sigma(T)\frac{E-E_0}{k_\mathrm{b}T}\right] \quad \text{(S13)}$$

where α is the absorption coefficient, α_0 and E_0 are constants, k_b is the Boltzmann constant, T is the temperature, E is the energy of the photon, and $\sigma(T)$ is the steepness parameter. From the theory developed by Dow and Redfield[12], the steepness parameter governs the strength of the carrier phonon coupling and can be written as

$$\sigma(T) = \sigma_0 \frac{2k_\mathrm{b}T}{\hbar\omega}\tanh\left(\frac{\hbar\omega}{2k_\mathrm{b}T}\right) \quad \text{(S14)}$$

where σ_0 is a constant representing the limit of σ at high temperature, and $\hbar\omega$ represents the energy of the principal interacting phonon mode. The Urbach energy is now given by

$$E_\mathrm{u} = k_\mathrm{b}T/\sigma(T) \quad \text{(S15)}$$

and was obtained from an optical model based on transmission data (see Supplementary note 2), and is depicted in Fig. 3a of the main text.

Supplementary note 7 | Absorption edge shifts in high magnetic fields

By utilising very high magnetic fields (up to 65 T) the r.m.s. radius of the 1s exciton at 2 K can be inferred from the diamagnetic shift coefficient using the following relation[13]

$$\Delta E_\mathrm{dia} = \frac{e^2}{8\,m_\mathrm{r}}\langle r^2\rangle B^2 = c_0 B^2 \quad \text{(S16)}$$

where σ is the diamagnetic shift coefficient, m_r is the in-plane reduced mass, r is the radial coordinate in the plane perpendicular to the applied magnetic field B. In this case no circular polarisers were used

to distinguish between left and right circularly-polarised light, and therefore the shift in the exciton absorption is solely due to the diamagnetic contribution.

Supplementary note 8 | Convergence tests of the optical spectrum (BSE+ G_0W_0 scissor)

Here, we present the convergence of the dielectric function with respect to the **k**-point grid used for the calculations. We highlight that, albeit the spectral line shape has not reached full convergence yet, the excitonic eigenvalues associated with the peaks are sufficiently converged. We also highlight that the dielectric function was only calculated for q=0.

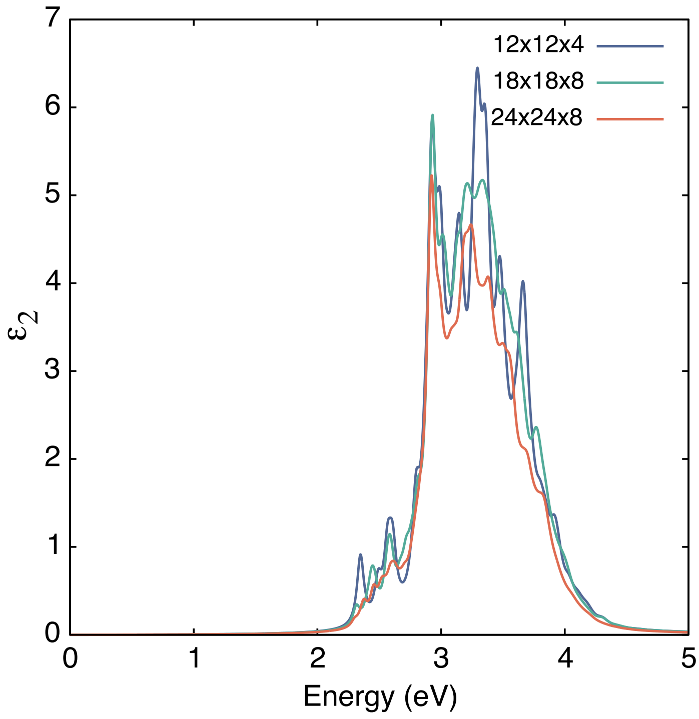

Supplementary Fig. 24 | Optical spectrum (BSE+G_0W_0) for BiOI. The spectrum is calculated with a 12×12×12, 18×18×6 and 24×24×8 **k**-point grid with a rigid scissor shift such as to reproduce the direct quasiparticle band gap. For each spectrum, four occupied and four unoccupied states enter the Bethe-Salpeter Hamiltonian.

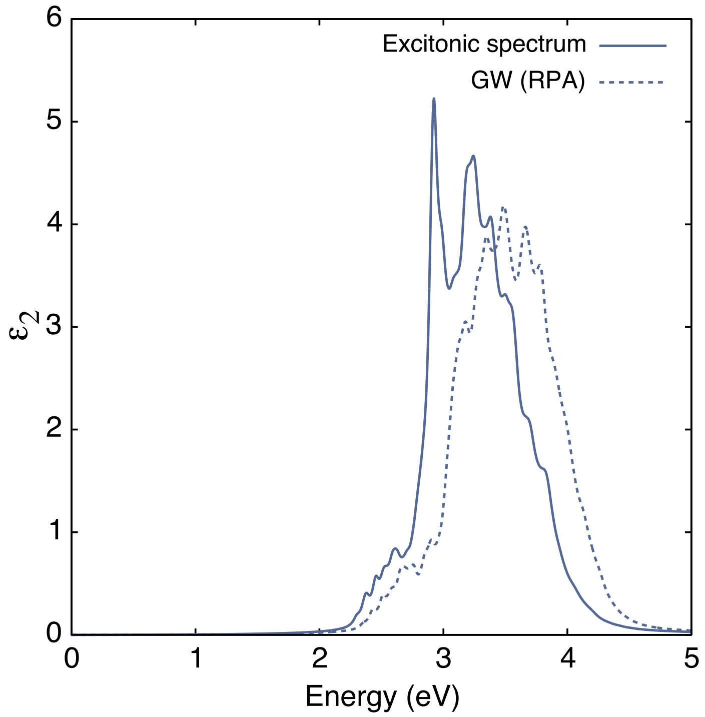

Supplementary Fig. 25 | Optical spectrum for BiOI. The spectrum is obtained from the BSE+G_0W_0 approach vs. single particle optical absorption obtained via the RPA (G_0W_0) formalism. Both spectra are calculated with a 24×24×24 **k**-point grid.

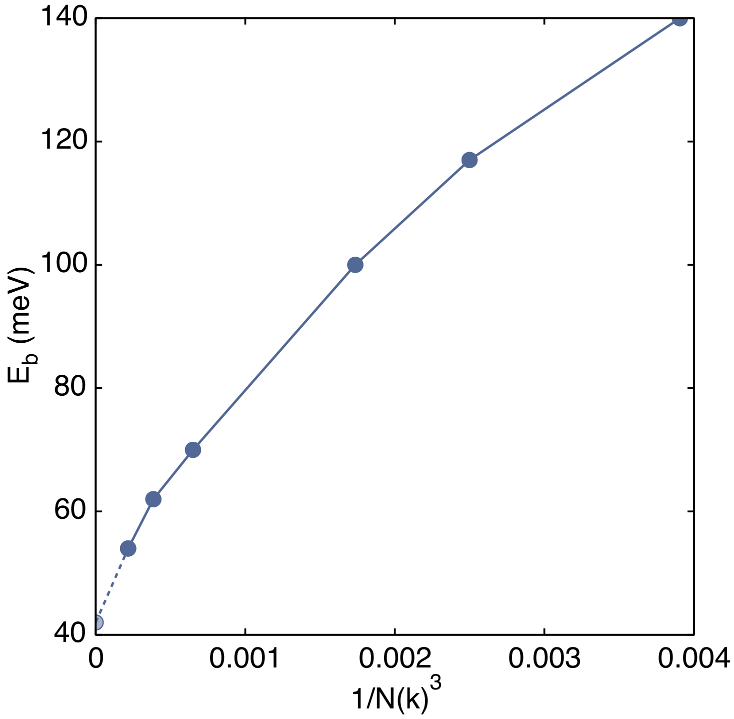

Supplementary Fig. 26 | Binding energy of the first bright exciton. As a function of **k**-point sampling grid size, where $N(k)^3$ is the number of **k**-points. We extrapolate the binding energy to an infinite **k**-point grid and estimate an exciton binding energy (without dynamic effects) of approximately 45 meV.

Supplementary note 9 | Structural distortion along Raman modes A and B

Fig 2d, right in the main text illustrates the vibrations along both Raman modes. For simplicity we only highlighted the main displacement vectors on the iodine and the bismuth for Raman mode A and B, respectively. However, pure iodine and bismuth out-of-plane vibrations are not eigenstates of the vibrational Hamiltonian. In Supplementary Fig. 27 we show all displacement vectors corresponding to the vibrational state A and B.

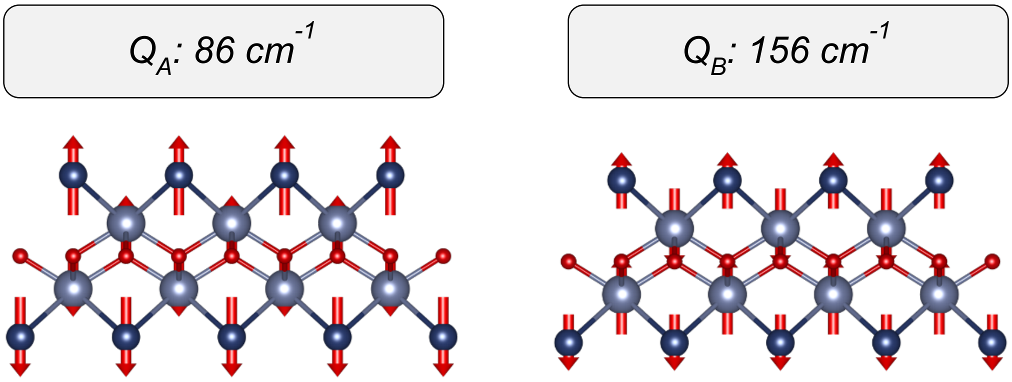

Supplementary Fig. 27 | Raman mode A and B. For Raman mode A, the displacement vectors on the Bi-atoms are enlarged. For Raman mode B, the displacement vectors on the I-atoms are enlarged. It is evident, that the vectors on the iodine are pointing into the same direction whilst the vectors on the Bi-atoms are pointing in opposite directions. Upon photoexcitation, both Raman-modes are activated and vibrate coherently, up to a configuration in which the Bi-atoms remain at the same position and the I-atoms moved away from the bismuth sublayer (new configuration in Supplementary Fig. 31).

The maximum displacements for both Raman modes (Q_A=10 and Q_B=10) correspond to the following displacement amplitudes:

Q_A^{max}

atom	x (Å)	y (Å)	z (Å)
Bi	0	0	0.0706382
Bi	0	0	-0.0706382
I	0	0	0.3312191
I	0	0	-0.3312191
O	0	0	0
O	0	0	0

Q_B^{max}

atom	x (Å)	y (Å)	z (Å)
Bi	0	0	- 0.2779016
Bi	0	0	0.2779016
I	0	0	0.0970678
I	0	0	-0.0970678
O	0	0	0
O	0	0	0

Supplementary note 10 | Potential energy surface

The small energy difference between the direct exciton and the indirect exciton generated upon photoexcitation make it very difficult to distinguish whether the photoluminescence arises from a direct or an indirect emission. In order to distinguish this, we calculate the potential energy surface of the lowest lying exciton (q=0) and compare it to the lowest lying exciton ($|q| \geq 0$) which we calculate solving the finite-q Bethe-Salpeter equation as it is implemented in Yambo. At the ground state geometry (Q_A=0/Q_B=0), the lowest lying exciton is, as expected, of indirect nature. From Supplementary Fig. 28, it becomes evident that the coupling between the indirect exciton and the Raman modes is weaker than for the blue surface up to the point where the direct exciton is the lowest lying state (for Q_A=9/Q_B=6).

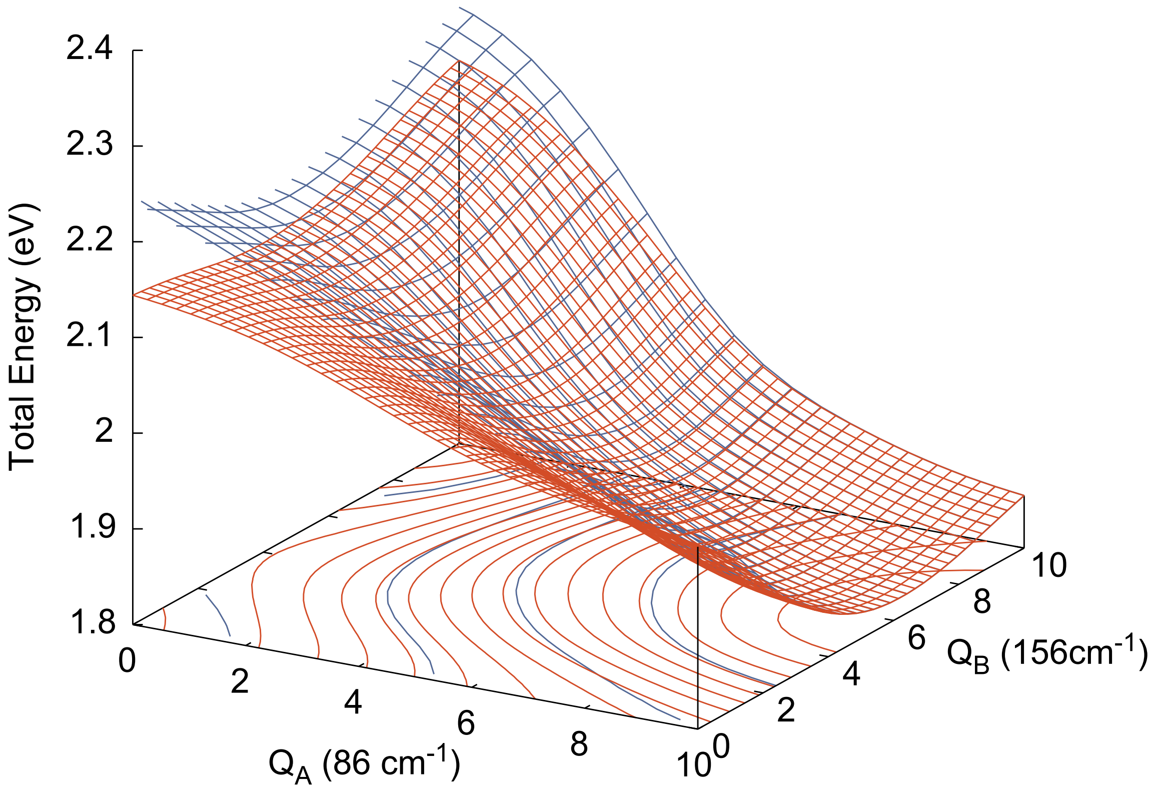

Supplementary Fig. 28 | Potential energy surface of the lowest lying (indirect) exciton and the lowest lying direct exciton. At the ground state equilibrium structure, the lowest lying exciton (red surface) is of indirect nature. However, the excitation of both the lowest lying direct exciton (blue) and the lowest lying indirect exciton leads to distortions along the totally symmetric Raman active modes (see Fig 2d in the main text) up to a point where the lowest lying exciton becomes direct.

We note that we cannot distort the system further than Q_A=10/Q_B=10, as it becomes metallic at the single-particle DFT level (due to the underestimation of the band gap). We presume, that due to the different curvatures of the ground- and the excited-state PES, we would approach a scenario in which a thermally-activated nonradiative decay is possible. However, due to the bandgap flaws in DFT (which serves as a starting point for the *GW* and Bethe-Salpeter calculation), the configurational sampling in the region of nonradiative decay remains prohibitive.

Supplementary note 11 | Exciton dispersion

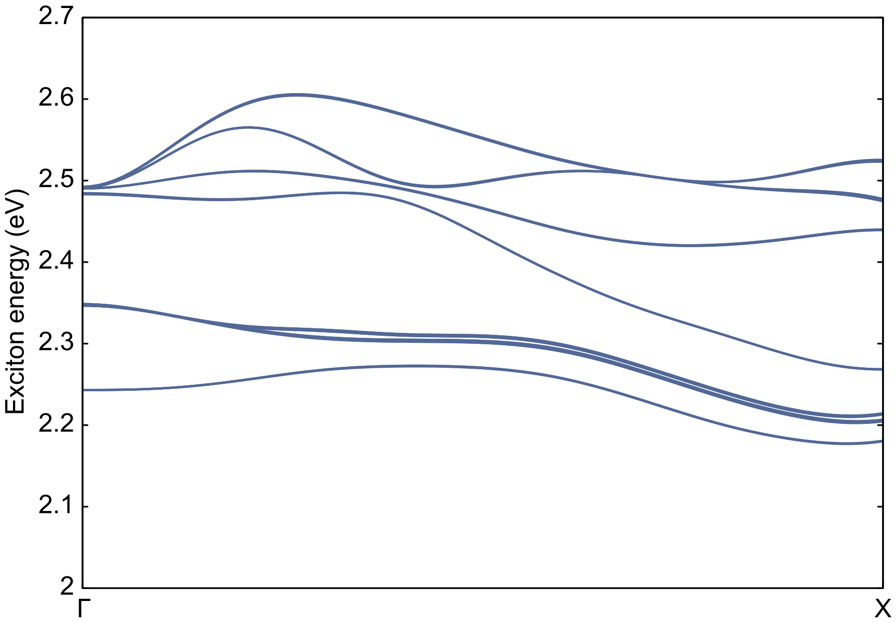

Supplementary Fig. 29 | Exciton dispersion of the ground state equilibrium structure. Dispersion of excitons for Q along Γ→X is obtained from the BSE+G_0W_0 approach with a 12×12×4 **k**-point grid.

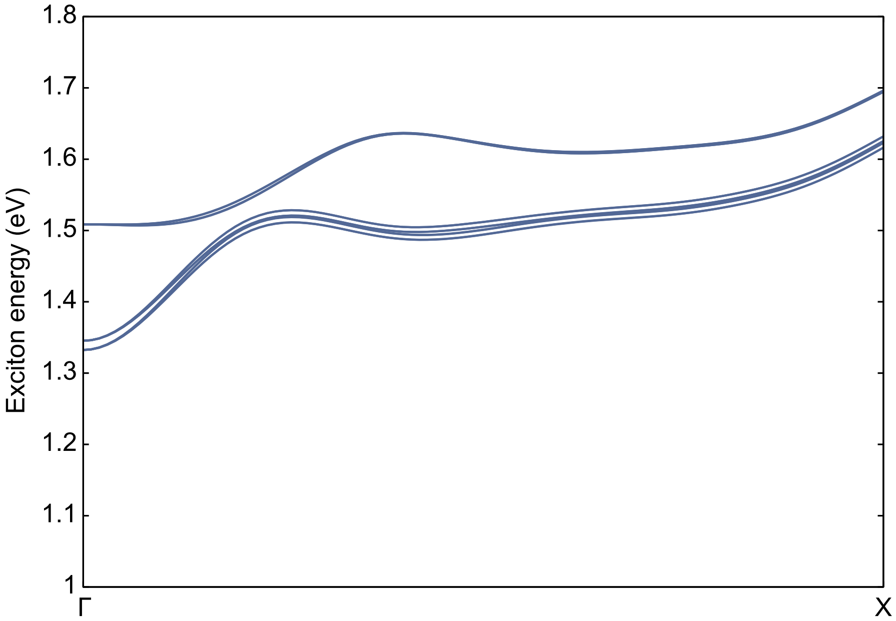

Supplementary Fig. 30 | Exciton dispersion of the excited state equilibrium structure. Dispersion of excitons for Q along Γ→X is obtained from the BSE+G_0W_0 approach with a 12×12×4 **k**-point grid.

Supplementary note 12 | Excited state wavefunctions.

In order to illustrate the retained delocalisation of the relaxed excited state we plot the electron and hole correlation to the exciton wavefunction for the equilibrium configuration of the 1s exciton.

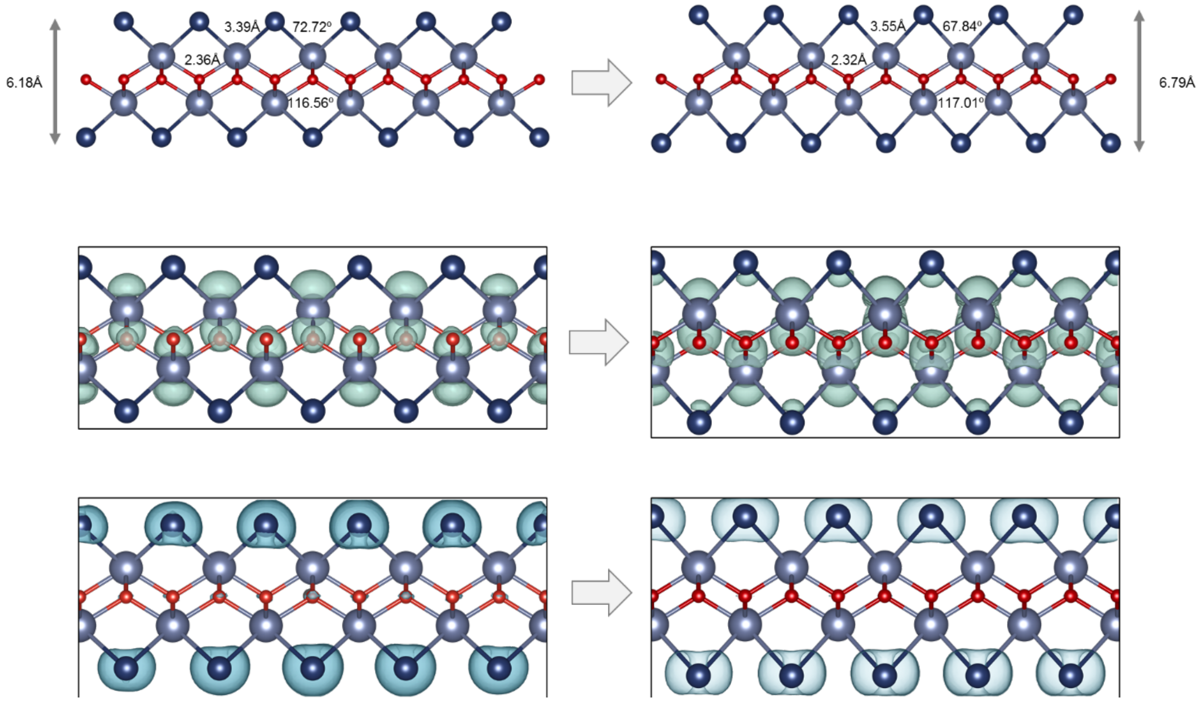

Supplementary Fig. 31 | Exciton maintains its delocalised features. On the left-hand side, we present the ground state equilibrium structure of BiOI prior to photoexcitation with the most important bond lengths and bond angles. Below, we illustrate the electron and hole densities of the first direct excitonic state. On the right-hand side, we depict the relaxed excited state structure (top) and the corresponding electron and hole densities of the excitonic wavefunctions (all isosurfaces are plotted with 20% to their maximum value). It is evident that the wavefunctions along the *xy*-plane remain delocalised. The bond lengths and angles were determined from DFT, and agree well with those we found experimentally, as shown in Supplementary Fig. 1.

Supplementary note 13 | Fröhlich coupling constant

In this section we calculate the Fröhlich coupling constant for a quantification of the electron-phonon coupling strength. This dimensionless constant α, albeit an approximation for isotropic materials, permits us to estimate the transport properties of BiOI and enables a quantitative comparison to other optoelectronic materials considered for similar applications.

The dimensionless Fröhlich constant (α) is determined by Equation S17:

$$\alpha = \frac{1}{2}\frac{1}{4\pi\varepsilon_0}\left(\frac{1}{\varepsilon_{\text{optical}}} - \frac{1}{\varepsilon_{\text{static}}}\right)\frac{e^2}{\hbar}\sqrt{\frac{2m^*}{\hbar\omega_{\text{LO}}}} \tag{S17}$$

where ω_{LO} is the LO phonon frequency, m^* the effective hole or electron mass, $\varepsilon_{\text{optical}}$ the high frequency dielectric constant, $\varepsilon_{\text{static}}$ the static dielectric constant, and ε_0 the permittivity of vacuum. We estimate the in-plane effective electron ($m_{\text{e}} = 0.23m_0$) and hole ($m_{\text{h}} = 0.26\ m_0$) mass (where m_0 is the rest mass of an electron), fitted from the band edges of the *GW* band structure. The static and high frequency dielectric constants are obtained using density functional perturbation theory[14–16] as it is implemented in the electronic structure code VASP. We note, that the dielectric constants have already been published elsewhere[17] ($\varepsilon_{\text{optical}} = 7.67$ and $\varepsilon_{\text{static}} = 68.61$) and that they significantly differ from the values obtained in this work ($\varepsilon_{\text{optical}} = 8.68$ and $\varepsilon_{\text{static}} = 43.21$). With additional exploration, we have found that this discrepancy is caused by the overestimation of the lattice parameters when the structural relaxation is conducted without a *van der Waals* correction. Since our lattice parameters are closer to the measured room-temperature values, we use the values calculated in this work to estimate the coupling constant α. We calculated the value of α for in-plane transport to range from 1.17 (using $\omega_{\text{LO}} = 4.68(2\pi)$ THz [156 cm^{-1}], $m^* = 0.23m_0$) to 1.68 (using $\omega_{\text{LO}} = 2.58(2\pi)$ THz [86 cm^{-1}], $m^* = 0.26m_0$).

Supplementary note 14 | Further details on performance of X-ray detectors

In the main text, we only showed the performance of BiOI detectors made in the perpendicular configuration. This is because: i) these devices had the lowest dark currents (Supplementary Fig. 10) that were stable over time, and ii) the devices are comprised of electrodes deposited onto the broad (00*l*) facets of the crystals that are large and parallel to each other, and through which we can easily directly illuminate with X-ray through one electrode. The importance of point ii) is that we can generate free carriers directly next to one electrode, which fulfils one of the assumptions of the modified Hecht equation, allowing for facile and robust extraction of the $\mu\tau$ products.

In the parallel configuration, measuring the $\mu\tau$ product is not as straightforward, owing to the highly anisotropic nature by which BiOI crystals grow. Whilst the (00*l*) facets are 1–5 mm in size, the cross-section is only 0.05-0.2 mm in size. This makes it impractical to deposit electrodes directly onto opposite (*h*00) or (0*k*0) planes, and it is also not practical to collimate the X-ray beam to only illuminate these narrow planes; we would always have X-rays spilling over to the (00*l*) face. Simply illuminating the full channel with X-rays while applying a field across the channel (Supplementary Fig. 32a, left) would not fulfil one of the assumptions held in the modified Hecht equation, since electrons and holes are generated across the entire channel and both charge-carriers are extracted. We therefore used a 400 µm thick Cu mask to cover 8/9 of the channel. The mask attenuated c. 95% of the incident X-rays. The 250 µm slit that is open has a width comparable to the depth for 35 keV X-rays in BiOI in which 90-99% of incident photons are attenuated. Thus, the setup shown in Supplementary Fig. 32a, right, provides conditions that approximately fulfil the assumptions held in the modified Hecht model, within practical constraints. Importantly, by generating free charge-carriers next to one electrode, we are able to determine the $\mu\tau$ product for only electrons or only holes, the selection of which is determined the polarity of the high terminal.

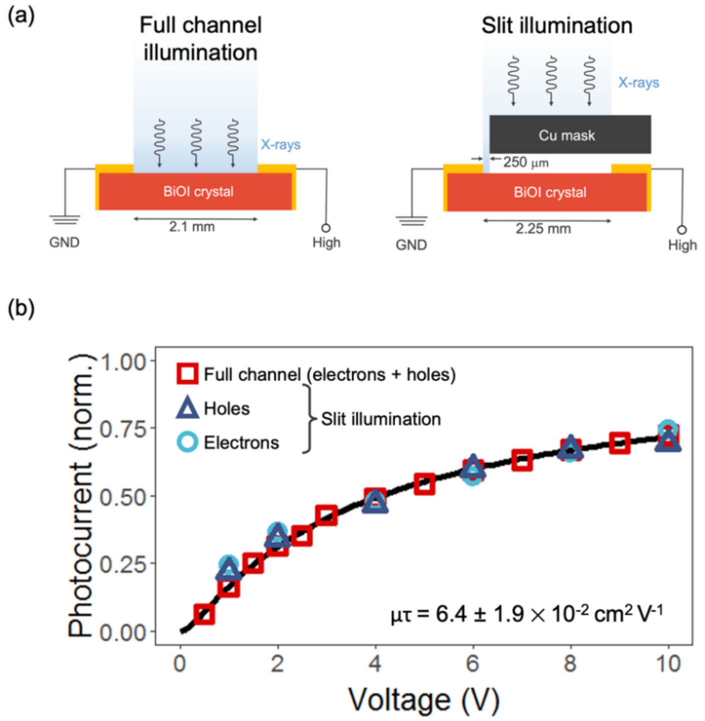

Supplementary Fig. 32 | Photocurrent from devices measured in the parallel configuration. a, Schematic of device measurement with full channel illumination (left) and illumination through a 250 µm-wide slit next to one of the electrodes (right). Note that the channels lengths are slightly different in the two cases because different samples were used, but these were both grown in the same conditions. **b,** Voltage-dependent photocurrent measurements of parallel BiOI devices measured with

full channel illumination (squares) *vs.* with slit illumination (triangles and circles). With full-channel illumination, electrons and holes generated at all points, and both charge-carriers are extracted from the electrodes. With the slit illumination method, all charge-carriers are generated next to one electrode and, depending on the polarity of the high terminal, only electrons or holes are extracted from ground, and it is for these carriers that the $\mu\tau$ product relates to.

In the parallel configuration, the $\mu\tau$ product is an order of magnitude larger than in the perpendicular configuration (1.1×10^{-3} cm^2 V^{-1}, see main text). However, we found the sensitivity to be higher in the perpendicular configuration at the same applied bias. This is because despite the order-of-magnitude higher $\mu\tau$ products in the parallel configuration, the transit distances were also an order of magnitude larger. The same applied bias would also produce an order of magnitude smaller electric field. Overall, these effects cancel out between the two configurations, as illustrated in Supplementary Table 4 for the case of 5 V bias (at which we measured sensitivities and LoDDs).

Supplementary Table 4 | Charge collection efficiencies of the parallel and perpendicular configuration devices for 5 V applied bias, estimated using the Hecht model

	Parallel	Perpendicular
μτ (cm^2 V^{-1})	6.4×10^{-2}	1.1×10^{-3}
Channel length (cm)	0.21	0.018
Electric field (V/cm)	23.8	278
CCE (%)	93.4	97.1

To understand the illustration in Supplementary Table 4 more intuitively, we can examine the Hecht model:

$$\mathrm{CCE} = \frac{\mu\tau E}{L}\left(1 - e^{-\frac{L}{\mu\tau E}}\right) \qquad \text{(S18)}$$

where CCE is the charge-collection efficiency, E the applied field (VL^{-1}) and L the transit length (which is taken to be the same as the distance between the electrodes). This applies to the case where electrons and holes are generated next to one electrode, and only one charge-carrier is transited to the other electrode. We can see that $\mu\tau E L^{-1}$ equals $\mu\tau V L^{-2}$. Thus, for the same applied bias, we can compare $\mu\tau L^{-2}$, which are 1.45 V^{-1} (parallel) and 3.40 V^{-1} (perpendicular). 1-exp(-[$\mu\tau V L^{-2}$]$^{-1}$) is then equal to 12.9×10^{-2} (parallel) and 5.7×10^{-2} (perpendicular). Thus, the product between $\mu\tau L^{-2}$, the applied bias V, and 1-exp(-[$\mu\tau V L^{-2}$]$^{-1}$) produces an overall CCE where the differences in these terms cancel out, producing CCE values that are very similar between the two configurations, with the perpendicular configuration overall having a slightly higher CCE, which would lead to higher sensitivities. Furthermore, the perpendicular configuration is a more ideal structure than the parallel configuration for radiation detector measurements because the active area is more precisely defined, and the dose rate of X-rays reaching the active part of the device can be more accurately calculated. We therefore only report the sensitivities and LoDDs for the perpendicular devices in this work.

Beyond CCE and sensitivity, reduced polarisability is another important advantage of the perpendicular configuration. The photocurrents at different applied biases are shown in Supplementary Fig. 32b. It can be seen that the photocurrents for electrons and holes are the same, and, intriguingly, are also similar to the photocurrents obtained from the full-channel illumination setup. Understanding the cause of this is beyond the scope of this current work. But fitting these data with the modified Hecht model gives a $\mu\tau$ product of $(6.4\pm1.9)\times10^{-2}$ cm^2 V^{-1}. As expected, this is larger than that found in the perpendicular configuration (Fig. 4c, main text) owing to the greater band dispersion in-plane than out-of-plane. This is also consistent with the time-of-flight measurements shown in Fig. 3d,e, main text, in which the maximum parallel mobility (83 cm^2 V^{-1} s^{-1}) exceeded the maximum perpendicular mobility (26 cm^2 V^{-1} s^{-1}).

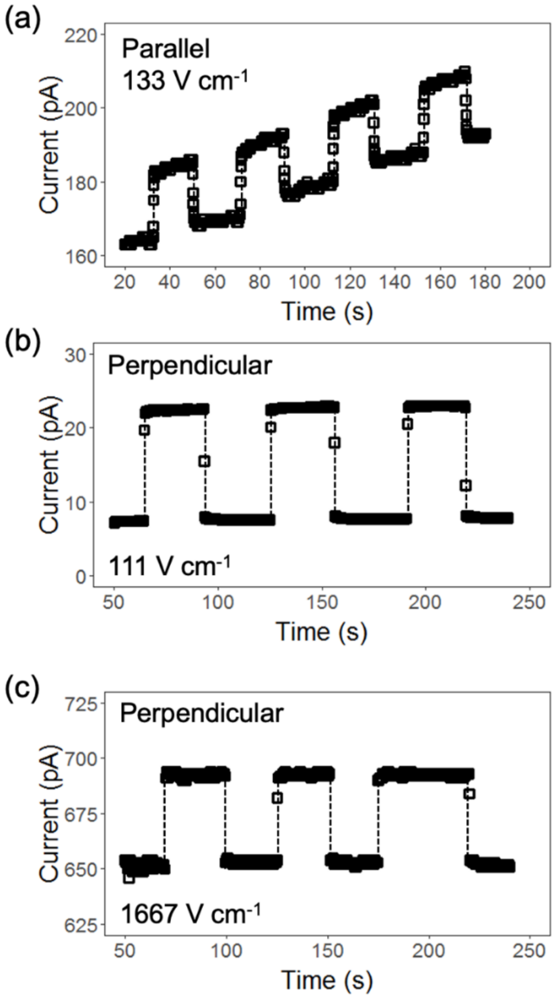

Supplementary Fig. 33 | Stability again ionic drift. Current *vs.* time traces of BiOI devices in the **a,** medium-field parallel (133 V cm^{-1} applied field; 30 V bias), **b,** medium-field perpendicular (111 V cm^{-1} applied field; 2 V bias), and **c,** high-field perpendicular (1667 V cm^{-1}, 30 V bias) configurations with and without X-ray illumination. From these measurements, it can be seen that there was ionic drift in the parallel devices only, given that the baseline current changed over time, whereas the perpendicular device baseline current remained steady over time.

We note that another important advantage of the perpendicular configuration was that we could bias these devices up to 30 V (1667 V cm^{-1}) without obtaining any baseline dark current drift, as shown in Supplementary Fig. 33c. By contrast, we found that the parallel devices started to exhibit dark current drift after applying 10 V bias (44 V cm^{-1}), as shown from the increase in the baseline dark current in Supplementary Fig. 33a. Dark current drift occurs due to ion migration in the bulk of the X-ray attenuation material[18]. To quantify the activation energy barrier to ion migration, we used a probe station to measure current at applied biases of 0 V and 7 V, with a biasing period of 80 s at each voltage. Each measurement was repeated three times. When the applied bias was switched to 0 V, we consistently observed the current immediately becoming negative and decaying towards the baseline (see Supplementary Fig. 34a for an example). This is consistent with previous observations widely made with lead-free perovskites and lead-halide perovskites[18–20], and is due to ions accumulated next to the electrodes attracting charge-carriers and causing drift in the opposite direction to when an external field

was applied. The decay in this negative current reflects the migration of ions back into the bulk of the material, and this was therefore the region fit to obtain the activation energy barrier. However, from a semilogarithmic plot of the normalised absolute current in this region, it is apparent that this is not a monoexponential decay (Supplementary Fig. 34a, inset). We therefore applied a tri-exponential model, i.e., $I = A_1 \exp(-t/\tau_1) + A_2 \exp(-t/\tau_2) + A_3 \exp(-t/\tau_3)$, to the normalised current that had the background current (original dark current before applying any bias) subtracted. This model was on the basis that there were three dominant migrating ionic species, which is consistent with multiple point defects (e.g., iodide vacancies, bismuth vacancies, and oxygen-on-iodine anti-sites) having low formation energies[21]. Following the approach taken previously by Pan *et al.*[18] and Duan *et al.*,[19] we took the reciprocal of the time constants (i.e., $k = 1/\tau$) to be proportional to ionic conductivity. The ionic conductivity is given by the model below[18]:

$$\sigma_{\mathrm{ion}} = \frac{Z_{\mathrm{i}} q^2 C_{\mathrm{V}}^0 D_{\mathrm{V}}^0}{k_{\mathrm{b}} T} \exp\left(-\frac{\Delta H_{\mathrm{S}}}{10 k_{\mathrm{B}} T}\right) \exp\left(-\frac{E_{\mathrm{a}}}{k_{\mathrm{b}} T}\right) \quad \text{(S19)}$$

where Z_{i} is the ionic charge, D_{V}^0 the diffusion coefficient, C_{V}^0 the ion/defect concentration, ΔH_{S} the formation energy of an ionic defect, E_{a} the diffusion barrier for ionic transport, and q the fundamental charge and k_{b} the Boltzmann constant. This equation can be simplified to:

$$\sigma_{\mathrm{ion}} T = \sigma_0 \exp\left(-\frac{E_{\mathrm{a}}^{\mathrm{eff}}}{k_{\mathrm{b}} T}\right) \quad \text{(S20)}$$

in which $E_{\mathrm{a}}^{\mathrm{eff}}$ is the effective activation energy barrier that accounts for both the formation and migration of the ionic defect. This activation energy barrier can therefore be obtained through a plot of $\ln(k_{\mathrm{b}} T)$ against $1/T$, which should give a linear plot with a slope of $-E_{\mathrm{a}}^{\mathrm{eff}}/k_{\mathrm{b}}$.

These Arrhenius plots are shown in Supplementary Fig. 34b–d for each of the three decay constants fit. It was found that for both the parallel and perpendicular devices, there was no correlation with inverse temperature for $\ln(k_1 T)$, suggesting that the early decay in current deviated from the assumptions made in the model fit. The parallel device also had no correlation for $\ln(k_2 T)$, but the perpendicular device showed a strong correlation. Both device configurations showed a correlation for $\ln(k_3 T)$. From these plots, we obtained $E_{\mathrm{a}}^{\mathrm{eff}} = 250\pm170$ meV for the parallel configuration, and $E_{\mathrm{a}}^{\mathrm{eff}}$ ranging from 350±50 meV to 420±140 meV for the perpendicular configuration (Supplementary Fig. 34c and d).

These effective activation energy barriers are qualitatively consistent with the observations made in Supplementary Fig. 33, in that there is less ion migration in the perpendicular configuration than the parallel configuration. This may be due to ionic drift between planes being more difficult. By contrast, in the in-plane direction, iodide vacancies, for example, have a low formation energy. There is also a large space between planes. As such, iodide vacancies may easily migrate along the top and bottom of each I–Bi–O–Bi–I layer. The effective activation energy barrier in the parallel devices of 250±170 meV is similar to those found in single crystals of $CsPbBr_3$ (228 meV)[22], but higher than $MAPbBr_3$ single crystals (126–168 meV)[19,23]. By contrast, the effective activation energy barrier in the perpendicular configuration exceeds those found in both perovskites, and is similar to or higher than that of $Cs_2AgBiBr_6$ single crystals measured via the same method (348 meV)[18]. This emphasises the benefits of using BiOI radiation detectors in the perpendicular configuration.

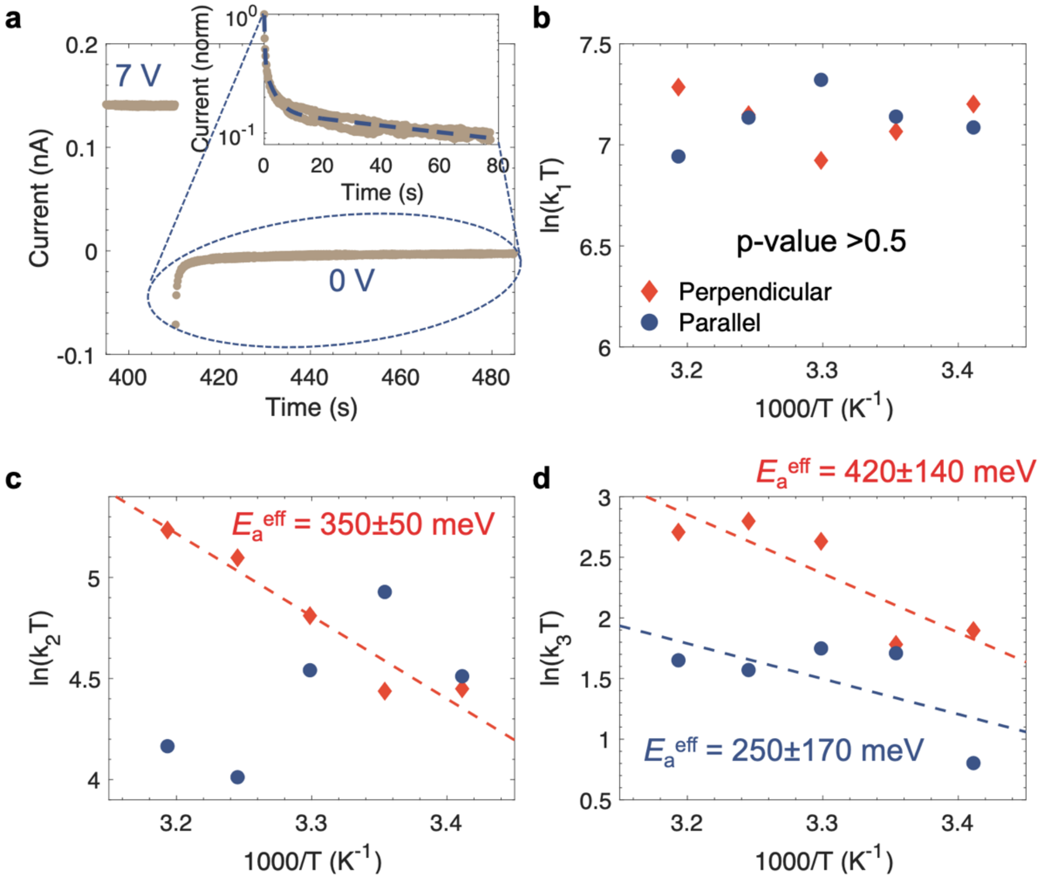

Supplementary Fig. 34 | Measuring the activation energy barrier for ion migration. a, Current *vs.* time as the applied bias was changed from +7 V to 0 V. Inset is the normalised current decay from the time when the voltage was switched to 0 V (time = 0 s). The triexponential decay model fit to the normalised current is shown as the dashed blue line. Measurement made for devices in the parallel configuration, but similar results were obtained from devices in the perpendicular configuration. Arrhenius plot of the natural logarithm of the product between the decay constant (k) and the absolute temperature (T) against inverse absolute temperature for the **b,** first, **c,** second and **d,** third decay constant for devices in the perpendicular (red) and parallel (blue) configuration. Dashed lines represent the Arrhenius model fit, where there was a trend.

A comparison between the performance of BiOI X-ray detectors in the perpendicular configuration with state-of-the-art detectors is given in Supplementary Table 5. The devices were measured with an applied bias of 5 V (278 V cm^{-1} field), which was chosen because this was at the inflection point in the photocurrent *vs.* applied bias plot (Fig. 4c, main text). An applied bias of 5 V therefore represents a compromise between high photocurrents *vs.* avoiding dark currents that are too high.

Supplementary Table 5 | Comparison of the key properties and performance of semiconductor X-ray detector materials

	a-Se	HgI_2	CZT	$MAPbBr_3$	BiOI (perp.)
Band gap (eV)	2.2	2.1	1.5-1.6	2.2	1.93
Atomic number	34	80,53	48,52	63.6	73.6
$\mu\tau$ product ($cm^2\ V^{-1}$)	10^{-7}	1.5×10^{-5}	4×10^{-3}-10^{-2}	1.2×10^{-2}	1.1×10^{-3}
Sensitivity ($\mu C\ Gy_{air}^{-1}cm^{-2}$)	20	1600	318	$2.1\text{x}10^4$	1100
Lowest detec-table dose ($\mu Gy_{air}s^{-1}$)	5.5	10	50	0.036	0.022[b]
Dark current density ($pA\ mm^{-2}$)[a]	0.1-8 (10 V μm^{-1})	10 (0.25 V μm^{-1})	90 (0.25 V μm^{-1})	230 (0.5 mV μm^{-1})	0.5 (5.5 V μm^{-1}))
Ref.	24, 25, 26	27, 28	29, 30, 31	32, 33	This work

[a] Electric field used for measuring the dark current reported shown in brackets

[b] The lowest dose rate directly measured was 22 $nGy_{air}\ s^{-1}$, but this had a signal-to-noise ratio (SNR) of 61, whereas the IUPAC standard takes the LoDD to be at an SNR of 3. Linearly extrapolating our measured dose rates down to SNR = 3 gives an estimated LoDD of 1.1 $nGy_{air}\ s^{-1}$.

N.B.: The X-ray ray energy source and applied field used to measure the detector performance reported in this table are not the same in all cases, which represent works spanning over two decades. Rather, the sensitivity and LoDD values represent the best-in-class. Details of the device structure, X-ray source used and applied field used to measure these parameters can be found in the references listed. However, the $\mu\tau$ products were extracted using the modified Hecht equation in most cases.

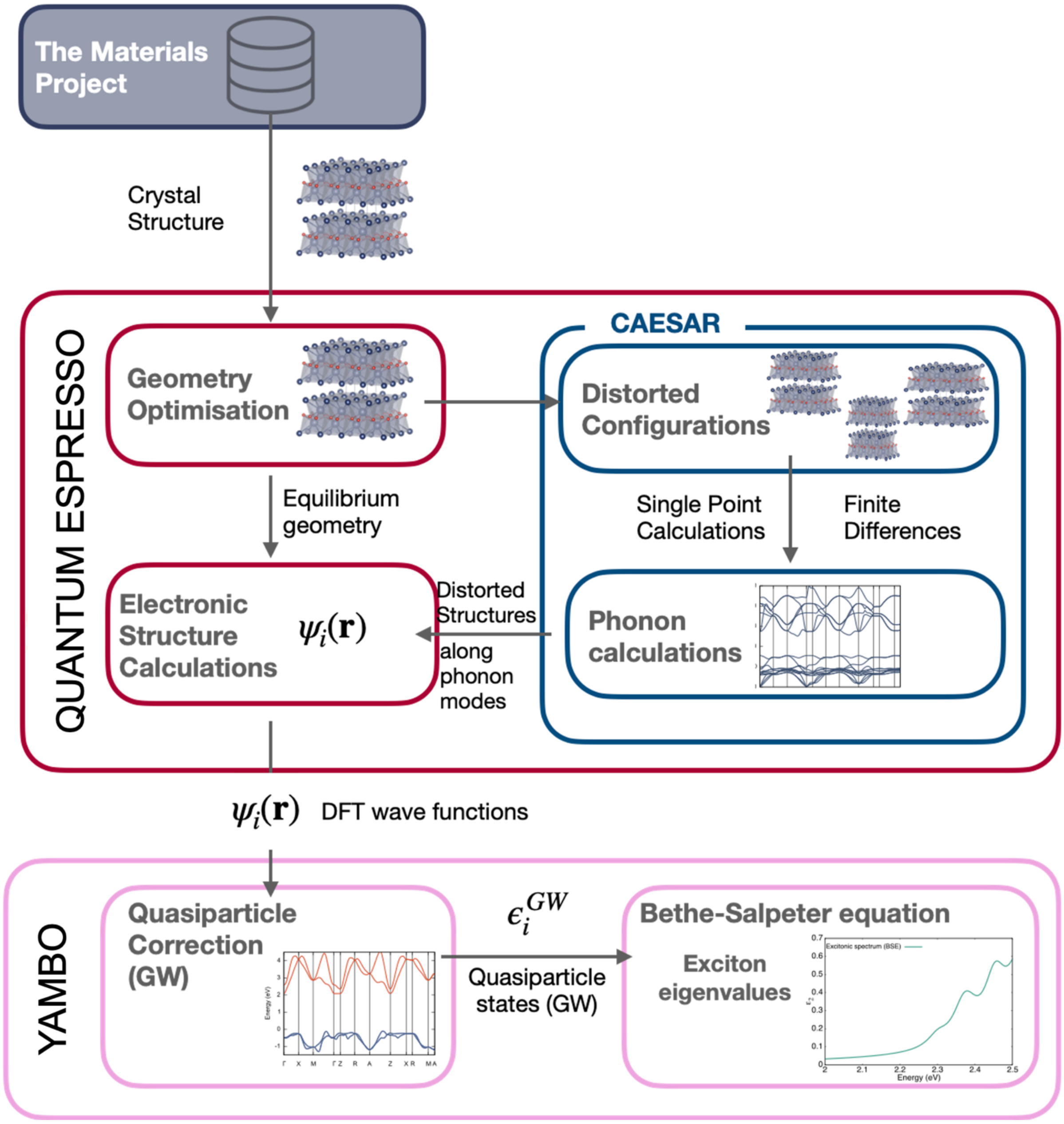

Supplementary Fig. 35 | Flowchart of the first-principles protocol employed in this work. The starting point of first-principles calculations is always the ground-state crystal structure, which was obtained from Materials Project. Using the crystal structure of BiOI we conducted a geometry optimisation using density functional theory as it is implemented in the open source electronic structure code QUANTUM ESPRESSO. The geometry optimisation was then followed by an electronic structure calculation, again using DFT. This gave us the mean-field electronic wavefunctions for the equilibrium structure. In parallel, we used the optimised geometry and conducted phonon calculations using finite differences with our in-house code CAESAR in conjunction with QUANTUM ESPRESSO. From the phonon calculations, we created distorted structures along the vibrational modes of interest (in this scenario the Raman-active modes observed in the TA measurement) and calculated the electronic wavefunctions for each. Then, the DFT wave functions were used for each configuration to calculate the quasiparticle corrections using the *GW* approximation as it is implemented in the electronic structure code YAMBO. The corrected electronic eigenvalues were used as the input to solve the Bethe-Salpeter equation to obtain the excitonic states and their eigenvalues for the equilibrium structure as well as the distorted configurations along the phonon modes studied.

References

1. Fabini, D. H. *et al.* Dynamic stereochemical activity of the Sn^{2+} lone pair in perovskite $CsSnBr_3$. *J. Am. Chem. Soc.* **138**, 11820–11832 (2016).
2. Schultz, P. & Keller, E. Strong positive and negative deviations from Vegard's rule: X-ray powder investigations of the three quasi-binary phase systems BiOX-BiOY (X, Y= Cl, Br, I). *Acta Crystallogr. B* **70**, 372–378 (2014).
3. Dong, X.-D., Yao, G.-Y., Liu, Q.-L., Zhao, Q.-M. & Zhao, Z.-Y. Spontaneous polarization effect and photocatalytic activity of layered compound of $BiOIO_3$. *Inorg. Chem.* **58**, 15344–15353 (2019).
4. Bannister, F. A. The crystal-structure of the bismuth oxyhalides. *Mineral. Mag. J. Mineral. Soc.* **24**, 49–58 (1935).
5. Saidaminov, M. I. *et al.* High-quality bulk hybrid perovskite single crystals within minutes by inverse temperature crystallization. *Nat. Commun.* **6**, 7586 (2015).
6. Taylor, M. L., Smith, R. L., Dossing, F. & Franich, R. D. Robust calculation of effective atomic numbers: The Auto-Z_{eff} software. *Med. Phys.* **39**, 1769–1778 (2012).
7. Berger, M. J. *et al.* NIST Standard Reference Database 8 (XGAM). (2010). https://dx.doi.org/10.18434/T48G6X
8. Born, M. & Wolf, E. *Principles of Optics: electromagnetic theory of propagation, interference and diffraction of light.* (Elsevier, 2013).
9. Lai, M. *et al.* Intrinsic anion diffusivity in lead halide perovskites is facilitated by a soft lattice. *PNAS* **115**, 11929–11934 (2018).
10. Hu, T. *et al.* Mechanism for broadband white-light emission from two- dimensional (110) hybrid perovskites. *J. Phys. Chem. Lett.* **7**, 2258–2263 (2016).
11. Furukawa, M., Mizuno, K., Matsui, A., Tamai, N. & Yamazaki, I. Branching of exciton relaxation to the free and self-trapped exciton states. *Chem. Phys.* **138**, 423–432 (1989).
12. Dow, J. D. & Redfield, D. Toward a unified theory of Urbach's rule and exponential absorption edges. *Phys. Rev. B* **5**, 594–610 (1972).
13. Stier, A. V, McCreary, K. M., Jonker, B. T., Kono, J. & Crooker, S. A. Exciton diamagnetic shifts and valley Zeeman effects in monolayer WS_2 and MoS_2 to 65 Tesla. *Nat. Commun.* **7**, 10643 (2016).
14. Baroni, S., Giannozzi, P. & Testa, A. Elastic Constants of Crystals from Linear-Response Theory. *Phys. Rev. Lett.* **59**, 2662–2665 (1987).
15. Baroni, S. *et al.* Phonons and related crystal properties from density-functional pertubation theory. *Rev. Mod. Phys.* **73**, 515–562 (2001).
16. Gonze, X. & Lee, C. Dynamical matrices, Born effective charges, dielectric permittivity tensors, and interatomic force constants from density-functional perturbation theory. *Phys. Rev. B* **55**, 355–368 (1997).
17. Ran, Z. *et al.* Bismuth and antimony-based oxyhalides and chalcohalides as potential optoelectronic materials. *npj Comput. Mater.* **4**, (2018).
18. Pan, W. *et al.* $Cs_2AgBiBr_6$ single-crystal X-ray detectors with a low detection limit. *Nat. Photonics* **11**, 726–732 (2017).
19. Li, D. *et al.* Electronic and ionic transport dynamics in organolead halide perovskites. *ACS Nano* **10**, 6933–6941 (2016).
20. Dong, Q. *et al.* Understanding the role of ion migration in the operation of perovskite light-emitting diodes by transient measurements. *ACS Appl. Mater. Interfaces* **12**, 48845–48853 (2020).
21. Hoye, R. L. Z. *et al.* Strongly enhanced photovoltaic performance and defect physics of air-stable bismuth oxyiodide (BiOI). *Adv. Mater.* **29**, 1702176 (2017).
22. Xia, M. *et al.* Unveiling the structural descriptor of $A_3B_2X_9$ perovskite derivatives toward X-ray detectors with low detection limit and high stability. *Adv. Funct. Mater.* **30**, 1910648 (2020).
23. Meloni, S. *et al.* Ionic polarization-induced current–voltage hysteresis in $CH_3NH_3PbX_3$

perovskite solar cells. *Nat. Commun.* **7**, 10334 (2016).
24. Kasap, S. O. X-ray sensitivity of photoconductors: application to stabilized a-Se. *J. Phys. D. Appl. Phys.* **33**, 2853 (2000).
25. Hunter, M., Belev, G., Kasap, S. & Yaffe, M. J. Measured and calculated K-fluorescence effects on the MTF of an amorphous-selenium based CCD x-ray detector. *Med. Phys.* **39**, 608–622 (2012).
26. Kasap, S. *et al.* Amorphous selenium and its alloys from early xeroradiography to high resolution X-ray image detectors and ultrasensitive imaging tubes. *Phys. status solidi B* **246**, 1794 - 1805 (2009).
27. Zentai, G. *et al.* Mercuric iodide and lead iodide x-ray detectors for radiographic and fluoroscopic medical imaging. in *Medical Imaging 2003: Physics of Medical Imaging* **5030**, 77–91 (International Society for Optics and Photonics, 2003).
28. Du, H. *et al.* Investigation of the signal behavior at diagnostic energies of prototype, direct detection, active matrix, flat-panel imagers incorporating polycrystalline HgI_2. *Phys. Med. Biol.* **53**, 1325 (2008).
29. Ivanov, Y. M. *et al.* The possibilities of using semi-insulating CdTe crystals as detecting material for X-ray imaging radiography. *Phys. status solidi* 840–844 (2003).
30. Bellazzini, R. *et al.* Chromatic X-ray imaging with a fine pitch CdTe sensor coupled to a large area photon counting pixel ASIC. *J. Instrum.* **8**, C02028 (2013).
31. Tokuda, S., Kishihara, H., Adachi, S. & Sato, T. Preparation and characterization of polycrystalline CdZnTe films for large-area, high-sensitivity X-ray detectors. *J. Mater. Sci. Mater. Electron.* **15**, 1–8 (2004).
32. Wei, H. *et al.* Sensitive X-ray detectors made of methylammonium lead tribromide perovskite single crystals. *Nat. Photonics* **10**, 333–340 (2016).
33. Wei, W. *et al.* Monolithic integration of hybrid perovskite single crystals with heterogenous substrate for highly sensitive X-ray imaging. *Nat. Photonics* **11**, 315–322 (2017).
